# Hybrid care engagement phenotypes and glycemic outcomes in diabetes: a cluster analysis across two health systems

**DOI:** 10.1093/jamia/ocag063

**Published:** 2026-05-06

**Authors:** Namuun Clifford, Kathryn E Kemper-McIsaac, Haoxiang Yu, Taylor Rapson, Urmimala Sarkar, Elaine C Khoong

**Affiliations:** School of Nursing, The University of Texas at Austin, Austin, TX 78701, United States; Division of General Internal Medicine, Department of Medicine, University of California San Francisco, San Francisco, CA 94115, United States; Division of Clinical Informatics and Digital Transformation (DoC-IT), Department of Medicine, University of California San Francisco, San Francisco, CA 94143, United States; Department of Epidemiology and Biostatistics, University of California San Francisco, San Francisco, CA 94158, United States; Department of Electrical and Computer Engineering, The University of Texas at Austin, Austin, TX 78712, United States; Department of Health Systems and Population Health, University of Washington, Seattle, WA 98195, United States; Division of General Internal Medicine, Department of Medicine, University of California San Francisco, San Francisco, CA 94115, United States; Division of Clinical Informatics and Digital Transformation (DoC-IT), Department of Medicine, University of California San Francisco, San Francisco, CA 94143, United States; Division of General Internal Medicine, Department of Medicine, University of California San Francisco, San Francisco, CA 94115, United States; Division of Clinical Informatics and Digital Transformation (DoC-IT), Department of Medicine, University of California San Francisco, San Francisco, CA 94143, United States

**Keywords:** Diabetes Mellitus Type 2, telemedicine, primary health care, cluster analysis, digital Health

## Abstract

**Objective:**

Prior studies often examine single telehealth encounter types or aggregate all digital care, overlooking how patients combine multiple digital and in-person modalities in hybrid care. To address this gap, we derived hybrid care engagement phenotypes and assessed sociodemographic differences and associations with glycemic control among adults with type 2 diabetes (T2DM).

**Methods:**

We conducted a retrospective cohort study of 10 671 adults with T2DM receiving primary care at an academic (UCSF) or safety-net system (SFHN) from April 2021 to March 2023. K-medoids clustering was applied to five encounter modalities (in-person, video, telephone visits; portal messages; unscheduled telephone calls) to derive four engagement phenotypes. We assessed sociodemographic differences using chi-square and Kruskal-Wallis tests and evaluated associations between phenotype and follow-up HbA1c control using logistic regression. We tested interactions with baseline HbA1c and estimated predicted probabilities using Tukey-adjusted contrasts.

**Results:**

Four phenotypes emerged per system: Digitally Engaged Multimodal, Traditional High Utilizers, Digitally Leaning (UCSF), Telephone Reliant (SFHN), and Low Digital. UCSF patients belonged to digitally forward phenotypes, whereas SFHN patients concentrated in traditional, lower-tech phenotypes. Among patients with uncontrolled diabetes, digitally forward phenotypes had 13-20 percentage points higher predicted probability of achieving control (UCSF: 56% Digitally Leaning vs 36% Traditional; SFHN: 53% Multimodal vs 40% Telephone).

**Discussion:**

Phenotypes varied by health system and sociodemographic factors, with modest, system-specific associations between digitally forward phenotypes and glycemic control among patients with uncontrolled diabetes. Findings underscore structural and sociodemographic inequities in hybrid care engagement and the need for proactive, tailored strategies to promote equitable hybrid care.

## Background and significance

Diabetes is one of the most prevalent and costly chronic conditions in the United States, affecting 1 in 10 Americans, with 90-95% of cases being Type 2 Diabetes Mellitus (T2DM).^1^ Racial and ethnic minorities, low-income populations, and rural residents face disproportionate burdens rooted in social drivers (eg, food insecurity, neighborhood disadvantage, healthcare access).^1^^,^^2^ Team-based primary care can mitigate these inequities through prevention of complications, continuity of care, and treatment adherence.^3–6^ Yet, barriers related to insurance, cost, and geography make access uneven, perpetuating disparities and underscoring the need for care models that reach underserved groups.^2^

Hybrid primary care models, which expanded rapidly during the COVID-19 pandemic, represent a fundamental transformation in healthcare delivery, enabling patients to engage through a mix of in-person and telehealth modalities (eg, video visits, telephone calls, portal messaging).^7–9^ While this shift has expanded opportunities to improve care continuity and reach, it has also magnified inequities in digital access and engagement, raising concerns that benefits of hybrid care may not be equitably realized across populations.^10^^,^^11^ Furthermore, little is known about how patients engage with these multimodal systems, or how new patterns of engagement influence T2DM outcomes. Prior work often examines single modalities or aggregate telehealth utilization, without accounting for the combined digital and in-person care patterns or their relationship to outcomes.^12–14^ However, modalities differ in accessibility and clinical utility, and patients’ preferences, digital access, literacy, and health system infrastructure can all influence which modalities are used. Comparing hybrid care engagement patterns across health systems with distinct digital infrastructure, patient populations, and telehealth implementation approaches can further reveal how system context and patient characteristics interact to shape modality use and health outcomes.

To address this gap, we applied an unsupervised machine learning approach (k-medoids clustering) separately within two San Francisco health systems to derive hybrid care engagement phenotypes among adults with T2DM across a two-year period (April 2021-March 2023). We use the term “phenotypes” to describe empirically derived subgroups that share distinct patterns of hybrid care utilization, consistent with prior applications of data-driven phenotyping to characterize digital health engagement behaviors.^15–17^ This approach enabled us to identify system-specific patterns of engagement and compare how different populations navigate hybrid care in the post-pandemic era. By examining both the sociodemographic composition of these phenotypes and their associations with glycemic outcomes, this study offers new insights into the equity implications of hybrid primary care for patients with T2DM.

## Objectives

We aimed to (1) identify hybrid care engagement phenotypes among adults with T2DM across two health systems; (2) examine differences in sociodemographic and clinical characteristics across phenotypes; and (3) evaluate associations with glycemic outcomes.

## Methods

### Study design and data source

This study used a retrospective cohort design to analyze hybrid primary care engagement and glycemic outcomes among 10 671 adults with T2DM (3860 academic; 6811 safety-net) across two urban San Francisco health systems. De-identified electronic health record (EHR) data comprising sociodemographic, clinical, and encounter variables were analyzed for patients receiving primary care at either the University of California, San Francisco (UCSF; an urban academic health system) or the San Francisco Health Network (SFHN; a public safety-net system) between April 1, 2021, and March 31, 2023. This time period was selected to capture the post-pandemic phase when hybrid care (in-person and telehealth) had been available and reimbursed across both systems for approximately nine months.^8^ This study was approved by the UCSF Institutional Review Board (IRB #20-31253).

### Study sample

The analytic cohort included adults ≥18 years who were empaneled in primary care as of April 1, 2019. Eligible patients were required to have at least one encounter with their care team during both the pre-study period (April 2019 to March 2021) and at least one healthcare interaction in the final 12 months of the study to ensure active engagement with the health system. Patients with type 1 or gestational diabetes, those with documented mortality during the study period, or those lost to follow-up (defined as no encounters in the final 12-month window), were excluded. Full eligibility criteria are provided in Supplementary Table S1.

### Variables and measures

Clustering variables included the total number of encounters across five modalities: (1) in-person office visits, (2) video visits, (3) telephone visits, (4) patient portal messages, and (5) unscheduled telephone calls. Unscheduled telephone calls were ones that occurred between visits with any member of the care team; both billable and nonbillable visits were included. Detailed encounter definitions and provider types are provided in Tables S2 and S3.

Patient characteristics comprised age (continuous/categorical), sex (male, female), race/ethnicity, preferred language (English, Spanish, Chinese, Other/Unknown), insurance type (Commercial, Medicare, Medicaid, Uninsured, Healthy Workers [city program providing coverage for certain home health workers]), neighborhood socioeconomic status (nSES, quintiles Q1-Q5), portal activation (active, inactive), and Charlson comorbidity index (CCI, score range: 0-16).^18^ Race and ethnicity were combined into a mutually exclusive derived variable (non-Hispanic White, Asian American, African American, Hispanic, Other), using institutional health equity guidance to create socially meaningful categories for disparities research. Neighborhood SES is an index including data on income, education, poverty, employment, occupation, housing, and rent values from American Community Survey 2013-2017 5-year estimates.^19^

Glycemic control was defined by hemoglobin A1c (HbA1c), categorized as controlled (≤8%) or uncontrolled (>8%) consistent with American College of Physicians’ guidance for T2DM.^20^ Baseline (October 2019-March 2021) and follow-up (October 2021-March 2023) HbA1c values were identified within 18-month windows to minimize missing data, as 12-month windows would have excluded ∼30% of patients without available labs. A sensitivity analysis using 12-month windows was also performed. Full definitions and coding are provided in Table S4.

### Analytic approach

#### Data preparation

We summarized patient characteristics using means and standard deviations (SDs) for approximately normally distributed continuous variables and frequencies and percentages for categorical variables, stratified by health system and engagement phenotype. Because encounter counts were right-skewed, we report both mean (SD) and median (interquartile ranges [IQRs]) for these variables. Encounter rates are reported per quarter to align with the standard 3-month follow-up interval recommended for diabetes management and HbA1c reassessment, providing a clinically meaningful unit of time for interpreting modality use patterns. Encounter counts were log-transformed and z-score normalized prior to clustering to address skewness and scale differences; zeros were retained as indicators of non-use. Additional preprocessing details are provided in Methods S1.1.

#### Cluster derivation

K-medoids clustering (Partitioning Around Medoids) with Manhattan distance was applied to each patient’s two-year totals across five encounter modalities: in-person, video, telephone, portal messages, and unscheduled telephone calls. Clustering was conducted separately by health system to account for system-level differences in telehealth implementation, as UCSF emphasized video visits while SFHN largely relied on telephone encounters.^8^ We evaluated solutions from *k* = 2 to 10 using silhouette widths and clinical interpretability.

#### Phenotype labeling and profiling

We labeled each phenotype by examining the relative distribution of modality use within clusters. Descriptive statistics guided our qualitative assessment of engagement patterns. We categorized video visits and portal messages as digitally forward modalities and in-person, telephone visits, and unscheduled telephone calls as traditional modalities. Using these categorizations, we applied labels that reflected dominant engagement patterns (eg, “Digitally Engaged Multimodal”, “Traditional High Utilizers”). We then profiled clusters by comparing patient characteristics across phenotypes using Pearson’s chi-square and Kruskal-Wallis tests.

#### Glycemic outcome analyses

We summarized baseline and follow-up HbA1c values, and their change, by engagement phenotype within each health system (Tables S5 and S6). We fit multivariable logistic regression models to estimate associations between engagement phenotypes and follow-up glycemic control (controlled ≤8% vs uncontrolled >8%), adjusting for baseline HbA1c status, age, sex, race/ethnicity, preferred language, insurance, and Charlson comorbidity index. Neighborhood SES (nSES) was summarized descriptively and incorporated into cluster profiling to characterize contextual differences across engagement phenotypes. nSES was not included in the adjusted regression models to maintain parsimony and because the primary aim of the regression analysis was to estimate associations between engagement phenotypes and glycemic control adjusting for key clinical and individual-level characteristics. Low Digital served as the reference phenotype. We tested interaction effects between phenotype and baseline glycemic status, reporting adjusted odds ratios (aORs) with 95% confidence intervals. Population-marginal predicted probabilities and Tukey-adjusted pairwise comparisons were estimated using the *emmeans* package.^21^ A complete case approach was used due to overall missingness <10% (Table S7), and sensitivity analyses restricted the cohort to patients with baseline and follow-up HbA1c measured within 12 months. All analyses were conducted in R version 4.3.2, using relevant packages (eg, *cluster, factoextra, car, emmeans*). Additional model diagnostics and sensitivity analyses are provided in the Methods S1.5 and S1.6, respectively.

## Results

### Cohort characteristics

The analytic cohort included 3860 academic (UCSF) and 6811 safety-net (SFHN) patients. Baseline characteristics differed substantially between the two systems, reflecting underlying sociodemographic and clinical differences (Table 1). UCSF patients were older (67 vs 62 years) and more often White (24% vs 11%) and Asian American (40% vs 35%), whereas SFHN had more Hispanic patients (34% vs 12%). English language preference was predominant at UCSF (82%), while Chinese (19%) and Spanish (38%) were more common at SFHN. Insurance and nSES also differed: UCSF patients were mainly commercially (44%) or Medicare (43%) insured, with 76% in the highest two nSES quintiles; in contrast, SFHN patients were more often insured through Medicaid (36%) or uninsured (13%), and just half lived in the highest two nSES quintiles. Portal activation was substantially higher at UCSF (88% vs 22%), as was baseline HbA1c control (72% vs 63%).

**Table 1.** Baseline characteristics of patients with T2DM at Academic (UCSF) and Safety Net (SFHN) health systems.

Characteristic	Academic	Safety Net
	(*n* = 3860)	(*n* = 6811)
Age, mean ± SD	66.5 ± 13.7	61.8 ± 11.6
Sex, *n* (%)		
Female	2091 (54%)	3581 (53%)
Male	1769 (46%)	3229 (47%)
Race/Ethnicity, *n* (%)		
Hispanic/Latine	480 (12%)	2330 (34%)
NH Asian American	1528 (40%)	2394 (35%)
NH African American	527 (14%)	1027 (15%)
NH White	941 (24%)	722 (11%)
Other/Unknown	384 (10%)	338 (5%)
Language preference, *n* (%)		
Chinese	296 (8%)	1324 (19%)
English	3154 (82%)	2931 (43%)
Spanish	298 (8%)	1901 (28%)
Other/Unknown	112 (3%)	655 (10%)
Health insurance, *n* (%)		
Commercial	1687 (44%)	43 (1%)
Healthy workers	n/a	972 (14%)
Medicaid	494 (13%)	2432 (36%)
Medicare	1639 (43%)	2363 (35%)
Uninsured	1 (0%)	859 (13%)
Unknown	39 (1%)	142 (2%)
Neighborhood SES Quintile, *n* (%)		
1 (Lowest)	179 (5%)	924 (14%)
2	250 (7%)	820 (12%)
3	467 (13%)	1558 (24%)
4	919 (25%)	1475 (22%)
5 (Highest)	1853 (51%)	1825 (28%)
Baseline patient portal activation status, *n* (%)		
Activated	3384 (88%)	1463 (22%)
Inactivated	476 (12%)	5348 (79%)
Charlson Comorbidity Score, mean ± SD	1.7 ± 2.29	1.04 ± 1.71
Baseline A1c control		
A1c Controlled (≤ 8%)	2790 (72%)	4267 (63%)
A1c Uncontrolled (> 8%)	745 (19%)	1973 (29%)
Missing	325 (8%)	571 (8%)

### Descriptive patterns of hybrid care engagement by modality

**Figure 1.**
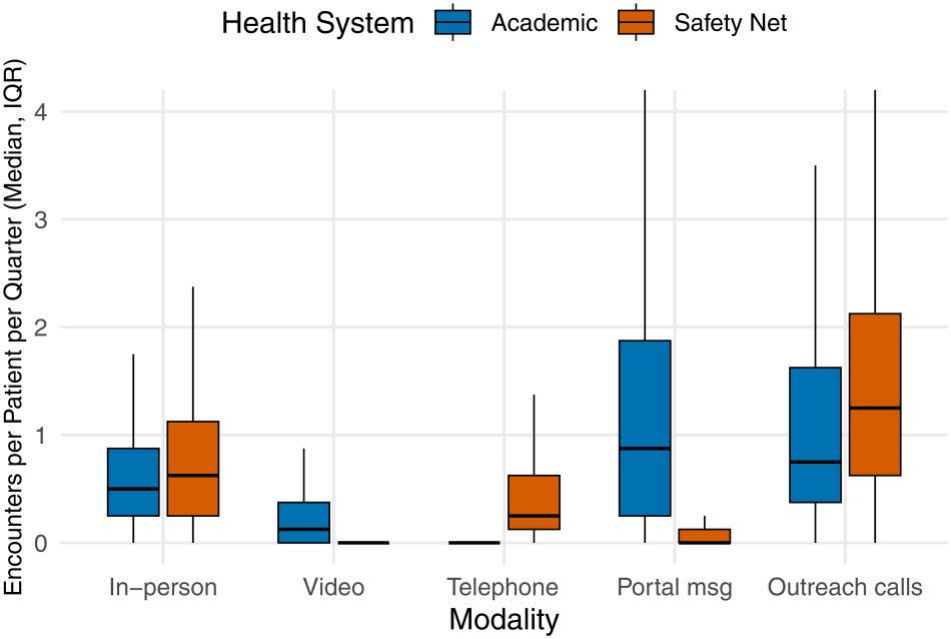
Quarterly encounter rates by modality and health system. Boxplots show the median and interquartile range (IQR) of encounters per patient per quarter across five modalities (in-person, video, telephone, portal messages, and unscheduled telephone calls) from April 2021 to March 2023, stratified by health system.

Hybrid care engagement patterns differed substantially between systems (Table 2; Figures 1 and S1). UCSF patients engaged more through digitally forward modalities like video visits and portal messages, while SFHN patients relied on lower-tech, traditional modalities such as telephone, in-person visits, and unscheduled telephone calls. Median quarterly rates further highlight these differences: UCSF patients had higher video visits (0.12 vs 0.00) and portal messages (0.88 vs 0.00), whereas SFHN patients had higher in-person (0.62 vs 0.50) and telephone visits (0.25 vs 0.00), and unscheduled telephone calls (1.25 vs 0.75). Table S8 and Figure S2 present total encounters and standardized per-100 patient rates across systems, illustrating overall differences in hybrid care delivery during the study period.

**Table 2.** Descriptives of quarterly encounters per patient by health system from April 2021 to March 2023.

	Academic (*n* = 3860)		Safety Net (*n* = 6811)	
Modality	Mean ± SD	Median (IQR)	Mean ± SD	Median (IQR)
In-person visits	0.65 ± 0.63	0.50 (0.25-0.88)	0.88 ± 1.01	0.62 (0.25-1.12)
Video visits	0.31 ± 0.44	0.12 (0.00-0.38)	0.01 ± 0.08	0.00 (0.00-0.00)
Telephone visits	0.06 ± 0.19	0.00 (0.00-0.00)	0.42 ± 0.49	0.25 (0.12-0.62)
Portal messages	1.41 ± 1.73	0.88 (0.25-1.88)	0.23 ± 0.83	0.00 (0.00-0.12)
Unscheduled telephone calls	1.32 ± 1.83	0.75 (0.38-1.62)	1.67 ± 1.61	1.25 (0.62-2.12)

Values are mean + SD and median (IQR) encounters per patient per quarter over the 2-year study period (April 2021-March 2023) across all modalities.

### Cluster identification and phenotypes

**Figure 2.**
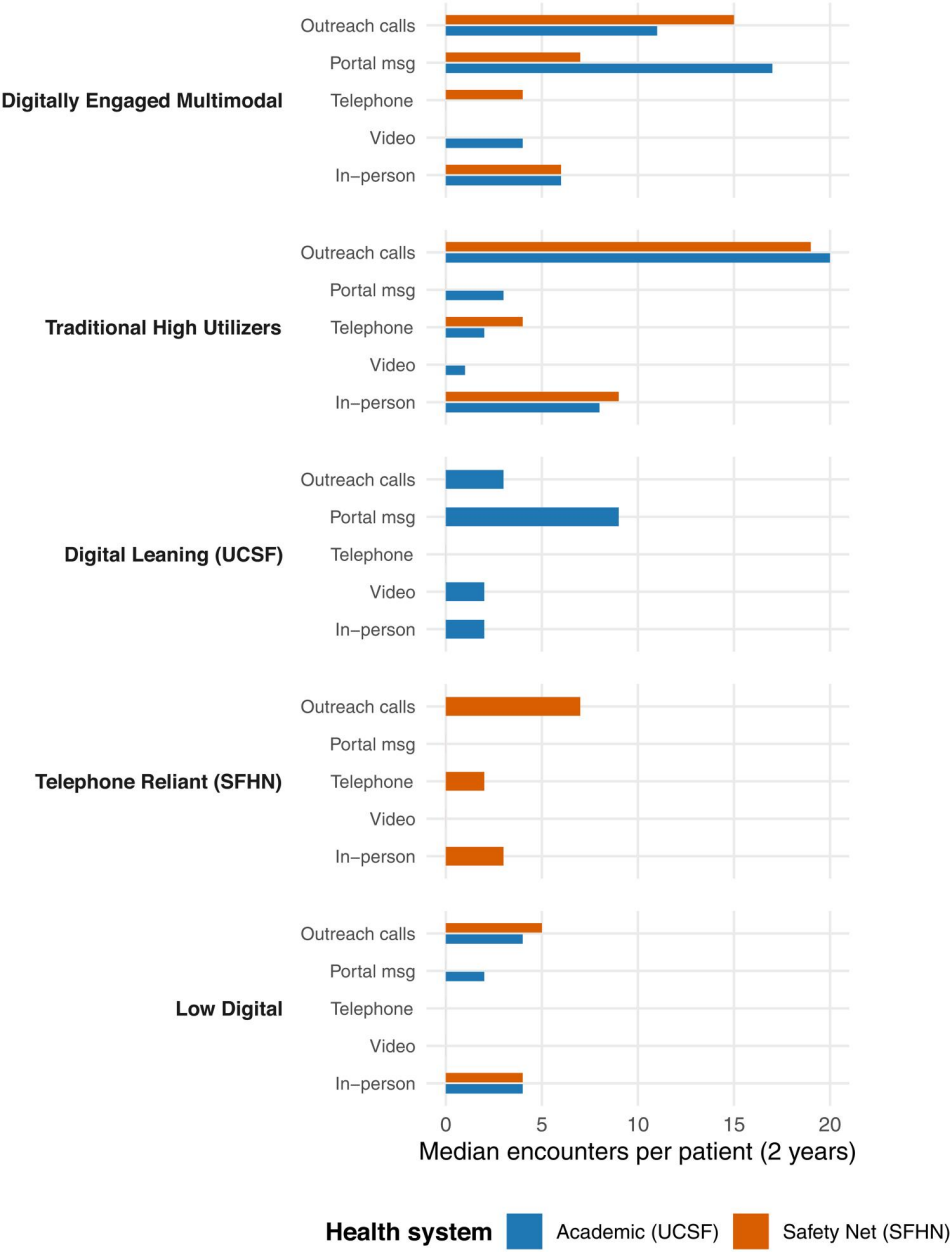
Median 2-year encounters by phenotype and health system. Horizontal bars show the median number of encounters per patient over the 2-year study period (April 2021-March 2023) across five modalities (in-person, video, telephone, portal messages, and unscheduled telephone calls), stratified by engagement phenotype and health system. Phenotype 3 differs by system: Digital Leaning at UCSF and Telephone Reliant at SFHN.

K-medoids clustering identified four distinct hybrid care engagement phenotypes within each health system (Table 3). While the 2-cluster solution yielded the highest silhouette width (0.25 at both sites), a 4-cluster solution (0.22) was selected because it produced behaviorally distinct, clinically interpretable phenotypes and showed good stability across multiple random starts (Table S9; Figure S3). Across systems, the four phenotypes reflect varying levels of digital versus lower-tech engagement (Figure 2).

**Table 3.** Median (IQR) encounter counts per patient by phenotype by health system.

Modality	Phenotype 1: Digitally Engaged Multimodal	Phenotype 2: Traditional High Utilizers	Phenotype 3: Digital Leaning (UCSF)/Telephone Reliant (SFHN)	Phenotype 4: Low Digital
Academic (*n* = 3860)				
*n* (%)	1124 (29%)	457 (12%)	1054 (27%)	1225 (32%)
In-person visits	6 (4-9)	8 (5-13)	2 (1-3)	4 (2-6)
Video visits	4 (2-7)	1 (0-2)	2 (1-3)	0 (0-0)
Telephone visits	0 (0-0)	2 (1-4)	0 (0-0)	0 (0--0)
Portal messages	17 (11-27)	3 (0-10)	9 (5-14)	2 (0-5)
Unscheduled telephone calls	11 (7-18)	20 (13-33)	3 (1-5)	4 (2-8)
Safety Net (*n* = 6811)				
*n* (%)	939 (14%)	2094 (31%)	2396 (35%)	1382 (20%)
In-person visits	6 (4-10)	9 (6-14)	3 (2-5)	4 (2-7)
Video visits	0 (0-0)	0 (0-0)	0 (0-0)	0 (0-0)
Telephone visits	4 (2-7)	4 (2-7)	2 (1-3)	0 (0-0)
Portal messages	7 (4-14)	0 (0-0)	0 (0-0)	0 (0-0)
Unscheduled telephone calls	15 (10-24)	19 (13-27)	7 (4-9)	5 (3-8)

Values are median (interquartile range) number of encounters per patient over the 2-year study-period (April 2021-March 2023). Phenotype 3 differs by health system: UCSF, Digital Leaning; SFHN, Telephone Reliant.

At UCSF (academic system; *n* = 3860), over half of patients belonged to digitally forward phenotypes—Digitally Engaged Multimodal (29%) and Digitally Leaning (27%)—characterized by greater use of video visits and portal messaging. In contrast, SFHN (safety-net system) was dominated by traditional and low-tech modalities, with two-thirds of patients classified as Traditional High Utilizers (31%) or Telephone Reliant (35%), reflecting greater reliance on in-person and audio-based encounters. Low Digital represented a smaller but notable share in both systems (20% safety-net, 32% academic).

In the academic system, the four phenotypes were: *Digitally Engaged Multimodal* (29%), with the highest use across most modalities, particularly portal messaging and video visits; *Traditional High Utilizers* (12%), relying heavily on in-person, telephone, and unscheduled phone calls but limited digital use; *Digitally Leaning* (27%), with moderate portal and video use and minimal in-person and unscheduled phone calls; and *Low Digital* (32%), with no video visits and few portal messaging encounters.

In the safety-net system (*n* = 6811), the four engagement phenotypes were: *Digitally Engaged Multimodal* (14%), combining high portal messaging use with frequent in-person, telephone, and unscheduled phone calls; *Traditional High Utilizers* (31%), relying mostly on in-person, telephone, and unscheduled telephone calls but no video or portal use; *Telephone Reliant* (35%), with low in-person frequency but moderate telephone visits and unscheduled telephone calls; and *Low Digital* (20%), with no video, portal messaging, or telephone encounters.

### Patient characteristics by phenotype

Sociodemographic and clinical characteristics varied significantly across engagement phenotypes within each health system (all *P*-values < .001; Tables S10-S12). Across systems, patient profiles varied by phenotype, with both shared and system-specific patterns. Digitally Engaged Multimodal patients in both systems were younger, English-language preferring, and lived in higher SES neighborhoods, with high portal activation and commercial or Medicare coverage. At UCSF, this phenotype included a higher proportion of Hispanic patients than the other phenotypes, whereas at SFHN, it included a higher proportion of non-Hispanic White patients.

Traditional High Utilizers were the oldest and most medically complex phenotype across both systems, with the highest comorbidity burden, lowest nSES and portal activation, greater proportions of African American and Spanish-language preferring patients, and the highest Medicare coverage. At UCSF, this group also had more Chinese language preference and Medicaid-insured patients, while at SFHN they included more Hispanic patients.

At UCSF, Digitally Leaning patients were the youngest, predominantly non-Hispanic White, English preferring, and commercially insured, with near-universal portal activation (98%) and highest nSES (76% in the top two quintiles).

At SFHN, the Telephone Reliant phenotype included the highest proportion of Asian American and Chinese-language preferring patients, more uninsured or Healthy Workers coverage, lower nSES, and minimal portal activation (16%).

Low Digital groups in both systems skewed older, non-English preferring, with lower portal activation. At UCSF, these patients were largely Asian American with higher nSES and commercial or Medicare coverage, whereas at SFHN they were more often Hispanic, Spanish-language preferring, lower nSES, Medicaid-insured, and had higher rates of missing baseline HbA1c.

### Glycemic outcomes by engagement phenotype

#### Academic health system (UCSF; n = 3860)

After exclusions, the analytic sample included 3241 patients (83.9% of initial cohort). Among patients with uncontrolled baseline HbA1c, predicted probability of achieving follow-up control ranged from 36% in Traditional High Utilizers to 56% in the Digital Leaning phenotype. Unadjusted means showed minimal change in HbA1c values for baseline-controlled patients and ∼1% reductions among baseline-uncontrolled patients, largest for Digitally Engaged Multimodal (-1.2%; Table S5). In adjusted main-effects models, phenotype was not independently associated with HbA1c control; baseline HbA1c and age were the strongest predictors (all *P*s < .01; Table S13).

For UCSF, adding a phenotype x baseline HbA1c interaction improved model fit (LRT *P* = .038; Table 4). Baseline uncontrolled HbA1c remained the dominant predictor (aOR = 0.09, 95% CI 0.06-0.14, *P* < .001), followed by older age compared to those <50 years (*P* < .001).

**Table 4.** Adjusted odds ratios (aOR) from the final interaction model predicting HbA1c control (≤8%) across both health systems.

	Academic (UCSF)		Safety Net (SFHN)	
Predictor	aOR (95% CI)	*P*-value	aOR (95% CI)	*P*-value
Engagement phenotype (ref = Low Digital)				
Digitally Engaged Multimodal	0.95 (0.67-1.33)	.75	1.25 (0.91-1.71)	.18
Traditional High Utilizers	0.95 (0.60-1.53)	.82	0.87 (0.67-1.12)	.28
Digitally Leaning	0.83 (0.59-1.15)	.27	—	—
Telephone Reliant	—	—	1.19 (0.92-1.54)	.19
Baseline HbA1c (ref = Controlled ≤8%)				
Uncontrolled >8%	0.09 (0.06-0.14)	**<.001**	0.14 (0.10-0.19)	**<.001**
**Age** (ref <50 years)				
50-64	1.51 (1.11-2.04)	**.009**	1.60 (1.32-1.94)	**<.001**
65-74	2.08 (1.48-2.94)	**<.001**	2.11 (1.67-2.66)	**<.001**
75+	1.69 (1.18-2.43)	**.004**	2.14 (1.59-2.90)	**<.001**
**Sex** (ref = Male)				
Female	1.06 (0.87-1.30)	.55	1.14 (0.99-1.30)	.06
Race/Ethnicity (ref = Non-Hispanic White)				
Hispanic/Latine	0.95 (0.66-1.36)	.76	1.12 (0.82-1.52)	.49
NH Asian American	1.16 (0.88-1.53)	.29	1.31 (1.00-1.72)	**.048**
NH African American	0.92 (0.65-1.29)	.61	1.45 (1.10-1.92)	**.008**
Other/Unknown	0.95 (0.67-1.37)	.78	1.25 (0.88-1.79)	.22
Preferred language (ref = English)				
Chinese	1.15 (0.74-1.82)	.53	1.19 (0.91-1.56)	.20
Spanish	1.17 (0.61-2.30)	.65	0.67 (0.52-0.87)	**.003**
Other/Unknown	0.83 (0.57-1.23)	.35	0.95 (0.72-1.24)	.69
Insurance (ref = Commercial UCSF/Medicare SFHN)				
Medicaid	0.86 (0.63-1.18)	.34	0.89 (0.74-1.07)	.23
Medicare	1.14 (0.88-1.46)	.32	—	—
Healthy Workers	—	—	1.25 (0.98-1.61)	.08
Uninsured/Unknown	—	—	1.11 (0.86-1.42)	.43
Charlson Comorbidity Index (ref = 0)				
1-2	1.19 (0.95-1.49)	.14	1.06 (0.91-1.23)	.44
3-4	1.18 (0.87-1.62)	.29	1.24 (0.96-1.60)	.11
5+	1.32 (0.92-1.91)	.14	1.12 (0.82-1.52)	.48
Interaction terms (ref = controlled HbA1c)				
Digitally Engaged Multimodal × Uncontrolled	1.22 (0.73-2.04)	.45	1.23 (0.77-1.96)	.39
Traditional High × Uncontrolled	0.69 (0.35-1.34)	.28	1.12 (0.76-1.65)	.58
Digitally Leaning × Uncontrolled	1.79 (1.01-3.16)	**.045**	—	—
Telephone Reliant × Uncontrolled	—	—	0.76 (0.51-1.13)	.17

**Figure 3.**
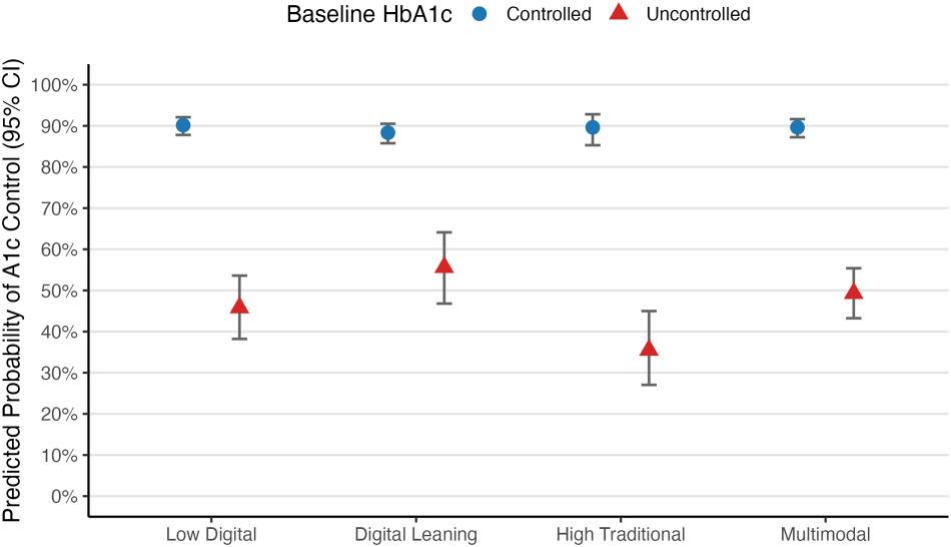
Adjusted predicted probability of follow-up HbA1c control by engagement phenotype and baseline HbA1c status at academic health system (UCSF). Points show population-marginal predicted probabilities of achieving HbA1c control (≤8%) at follow-up, stratified by engagement phenotype and baseline HbA1c status (controlled vs uncontrolled). Error bars indicate 95% confidence intervals. Estimates are adjusted for age, sex, race/ethnicity, preferred language, insurance, and Charlson comorbidity index.

**Figure 4.**
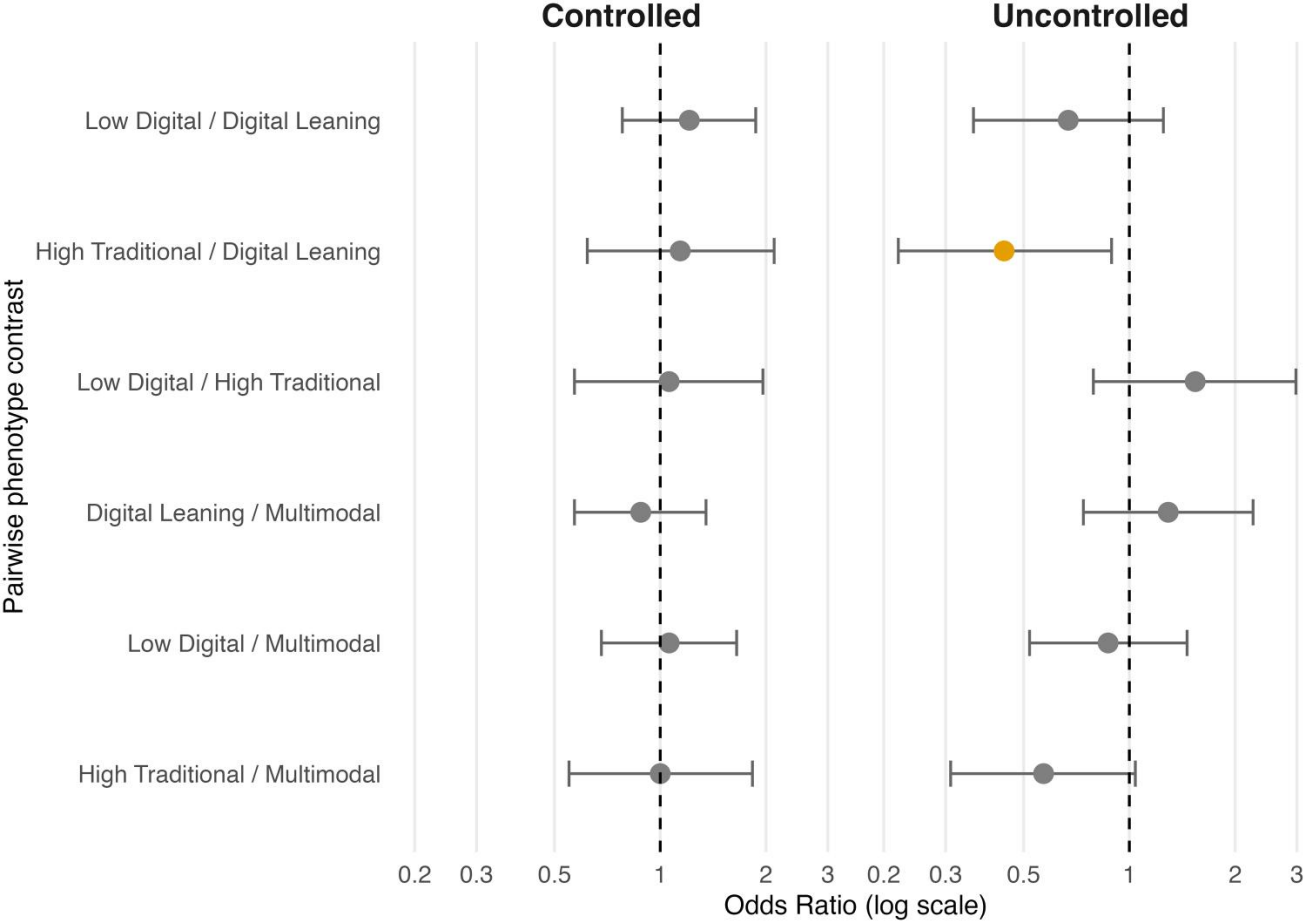
Pairwise odds ratios for HbA1c control by engagement phenotype (baseline uncontrolled HbA1c, UCSF). Points represent adjusted odds ratios for achieving HbA1c control (≤8%) from pairwise phenotype contrasts, stratified by baseline HbA1c status (controlled vs uncontrolled). Horizontal lines indicate 95% confidence intervals; the dashed vertical line denotes the null (OR = 1). Models adjust for age, sex, race/ethnicity, preferred language, insurance, and Charlson comorbidity index.

Among baseline-uncontrolled patients, Digital Leaning phenotype had higher odds of control than Low Digital (aOR = 1.79, 95% CI 1.01-3.16, *P* = .045). Population-marginal predicted probabilities of control were 46% Low Digital, 56% Digital Leaning, 49% Digitally Engaged Multimodal, and 36% Traditional High Utilizers (Figure 3, Table S14). Tukey adjusted pairwise contrasts showed Traditional High Utilizers had lower odds of HbA1c control than Digital Leaning (aOR = 0.44, 95% CI 0.22-0.89, *P* = .014; Figure 4). The model demonstrated good discrimination and calibration (AUC = 0.78; Hosmer-Lemeshow *P* = .75). See Figures S4 and S5.

#### Safety-net health system (SFHN; n = 6811)

**Figure 5.**
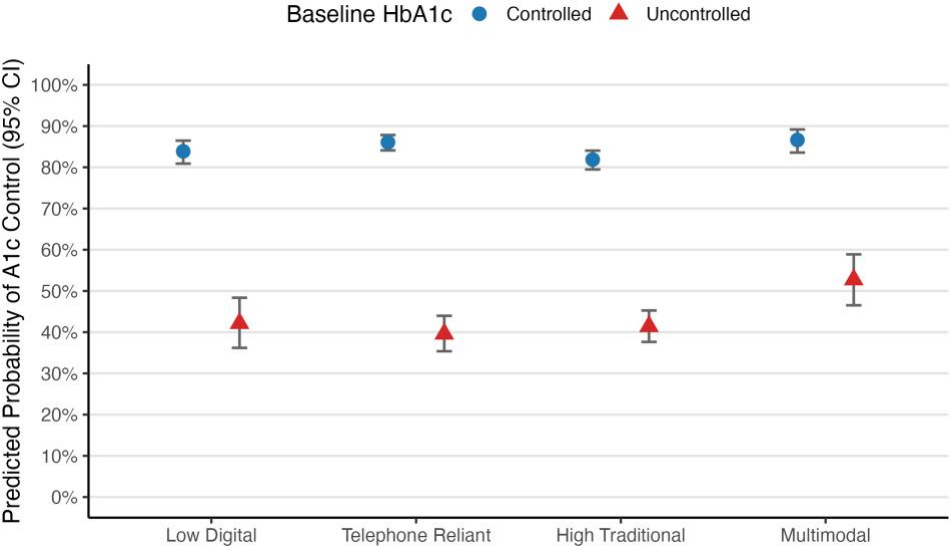
Adjusted predicted probability of follow-up HbA1c control by engagement phenotype and baseline HbA1c status at safety-net system (SFHN). Points show population-marginal predicted probabilities of achieving HbA1c control (≤8%) at follow-up, stratified by engagement phenotype and baseline HbA1c status (controlled vs uncontrolled). Error bars indicate 95% confidence intervals. Estimates are adjusted for age, sex, race/ethnicity, preferred language, insurance, and Charlson comorbidity index.

The analytic sample included 5751 patients (84.4%). Among baseline-uncontrolled patients, predicted probability of control ranged from 40% in Telephone Reliant to 53% in Digitally Engaged Multimodal (Figure 5, Table S15). Unadjusted mean reductions ranged from 0.7% to 1.2%, largest for Digitally Engaged Multimodal (Table S6). In main effects models, baseline HbA1c, age, race/ethnicity, and language were significant predictors (all *P*s <0.05; Table S13). Digitally Engaged Multimodal showed higher odds of HbA1c control compared with Low Digital (aOR 1.38, 95% CI 1.09-1.76, *P* = .008).

**Figure 6.**
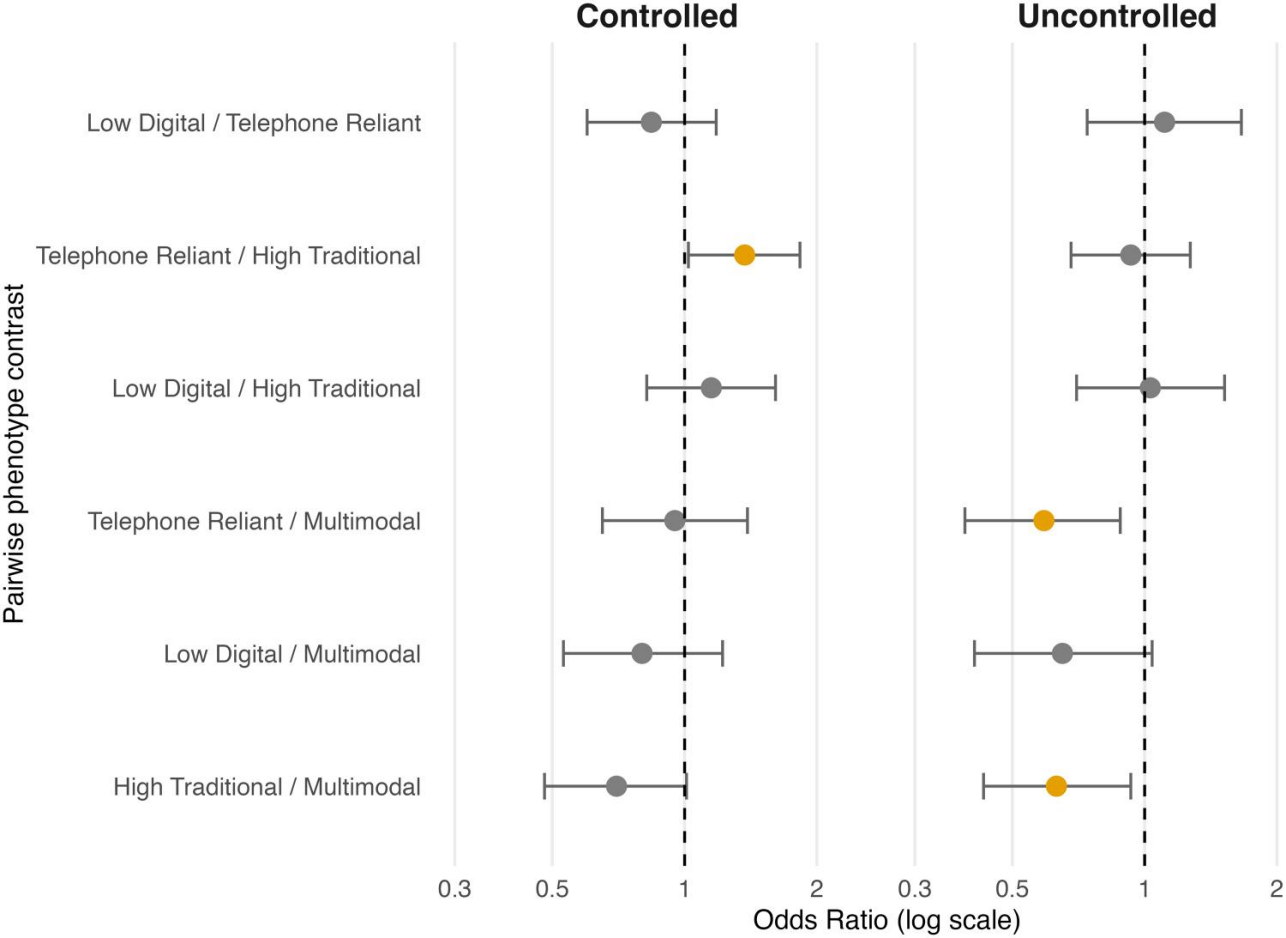
Pairwise odds ratios for HbA1c control by engagement phenotype (baseline uncontrolled HbA1c, SFHN). Points represent adjusted odds ratios for achieving HbA1c control (≤8%) from pairwise phenotype contrasts, stratified by baseline HbA1c status (controlled vs uncontrolled). Horizontal lines indicate 95% confidence intervals; the dashed vertical line denotes the null (OR = 1). Models adjust for age, sex, race/ethnicity, preferred language, insurance, and Charlson comorbidity index.

For SFHN, an interaction model improved fit (LRT *P* = .049). Baseline uncontrolled HbA1c remained the strongest predictor (aOR = 0.14, 95% CI 0.10-0.19, *P* < .001). Phenotype interactions were not statistically significant, and the Digitally Engaged Multimodal main effect attenuated (aOR = 1.25, *P* = .18). Older age, Asian and African American race, and English language preference were associated with higher odds of control, whereas Spanish language preference was associated with lower odds (Table 4). Pairwise Tukey-adjusted contrasts showed Digitally Engaged Multimodal had higher odds of control than Telephone Reliant (aOR = 1.69, 95% CI 1.14-2.56, *P* = .004) and Traditional High Utilizers (aOR = 1.59, 95% CI 1.08-2.33, *P* = .012; Figure 6). Model performance was good (AUC = 0.79; Hosmer-Lemeshow *P* = .54). See Figures S6 and S7.

Sensitivity analyses using the 12-month window (UCSF *n* = 2671; SFHN *n* = 4785) yielded consistent findings: baseline HbA1c remained the dominant predictor, age effects were unchanged, and SFHN phenotype and sociodemographic associations were directionally consistent though some attenuated.

## Discussion

### Principal findings

This study advances biomedical informatics by demonstrating how unsupervised clustering methods applied to routinely collected EHR encounter data can identify data-driven hybrid care engagement phenotypes. Using multimodal encounter data from 10 671 adults with T2DM across two urban health systems, we identified four behaviorally distinct hybrid care engagement phenotypes in each system that characterize how patients combine in-person, telephone, video, and portal-based care in the post-pandemic period. The phenotypes varied substantially by system and sociodemographic characteristics and showed modest, system-specific associations with glycemic outcomes among patients with uncontrolled diabetes. These findings demonstrate that hybrid care is not a uniform model but a set of heterogeneous care pathways, and that routinely collected encounter data can support identification of patient subgroups who may benefit from targeted outreach or digital support. By moving beyond single-modality telehealth metrics to a multimodal, phenotype-based framework derived from structured EHR data, we provide a reproducible analytic strategy for health systems seeking to monitor equity in hybrid care delivery.

### System- and patient-level drivers of hybrid care engagement

Hybrid care engagement differed markedly between systems. At the academic system, more than half of patients were in digitally forward phenotypes (Digitally Engaged Multimodal, Digital Leaning), reflecting strong uptake of video visits and portal messaging.

In contrast, two-thirds of safety-net patients clustered into Traditional or Telephone Reliant phenotypes, reflecting limited availability and adoption of video or portal-based modalities. These differences reflect divergent telehealth implementation trajectories during the pandemic, with UCSF adopting a video- and portal-centric model and SFHN emphasizing audio-based telehealth, a pattern common in safety-net settings.^8^^,^^22^^,^^23^ Although prior work emphasizes the role of age, race/ethnicity, language, and digital access in shaping telehealth use,^24–27^ our findings support growing evidence that system-level infrastructure, reimbursement policies, workflow integration, and technology support play a critical role in determining which hybrid care pathways are available and routinely used.^12^^,^^28–31^

Sociodemographic differences further shaped engagement. Digitally forward phenotypes were more common among younger, White, English-preferring patients residing in higher-SES neighborhoods with high portal activation. Low Digital and Traditional High Utilizer groups included more older adults, racial/ethnic minorities, non-English speakers, and individuals living in lower-SES neighborhoods with limited portal use. In the safety-net system, the Telephone Reliant phenotype disproportionately included Asian American and Chinese-language preferring patients with low portal activation, illustrating how language preference and system contexts influence modality use. These findings mirror well-documented digital divides: older adults, racial and ethnic minorities, individuals with limited device or broadband access, and those with lower digital literacy or limited English proficiency are consistently less likely to use digital health tools.^30^

### Engagement phenotypes and glycemic outcomes

Phenotype-outcome associations were modest and system-specific. In both systems, baseline HbA1c status was the dominant predictor of follow-up glycemic control. Among patients with uncontrolled baseline HbA1c, the predicted probability of achieving control ranged from 36% in the Traditional High Utilizers to 56% in the Digital Leaning phenotype at UCSF, and from 40% in the Telephone Reliant to 53% in the Digitally Engaged Multimodal at SFHN. In the safety-net system, Spanish language preference was independently associated with lower odds of glycemic control (aOR = 0.67; *P* = .003), consistent with prior literature findings that language barriers and language-discordant care contribute to glycemic disparities among Latino patients with limited English proficiency.^32^

Most chronic disease research still treats modality as a binary exposure (telehealth vs in-person), with limited work examining combinations of care modalities and their impact on disease outcomes. Among the few studies evaluating the differences between telehealth, in-person, and hybrid care utilization, results are inconsistent, with some studies finding comparable glycemic outcomes, while others finding telemedicine-only care had lower HbA1c improvement compared to in-person or hybrid care.^33^^,^^34^ In our study, baseline HbA1c status remained the primary predictor of glycemic control in both systems, and the absence of large phenotype-driven differences may reflect effective care delivery across modalities, unmeasured confounding, or both. These findings underscore the need for longitudinal research examining how multimodal engagement patterns, rather than individual modality comparisons, influence disease outcomes, which is more reflective of real-world healthcare utilization patterns. Future work should also examine how different engagement phenotypes impact adherence to guideline-recommended diabetes care processes (eg, foot examination, retinal and nephropathy screening), as well as downstream outcomes including emergency room visits, hospitalization, and mortality.

### Implications for hybrid care equity

These results have important implications for equitable hybrid care delivery. The stark contrast in modality availability across systems highlights how institutional infrastructure, reimbursement environments, and workflow design shape which hybrid care pathways are available to patients. In our study, engagement phenotypes were shaped primarily by system context and sociodemographic characteristics, underscoring that engagement with specific modalities is not solely patient-driven but structurally mediated. Prior evidence suggests that video visits may facilitate more comprehensive assessment, diagnostic accuracy, and improved communication compared with audio-only encounters.^35–37^ Although audio-only telehealth remains a vital access point for patients facing digital barriers, disproportionate reliance on telephone encounters may limit access to benefits embedded in more digitally enabled modalities.

Ensuring equitable access to all modalities is therefore essential to avoid creating “tiers” of hybrid care with potentially unequal clinical benefit. While reimbursement for audio-only visits remains essential particularly in safety-net settings, it must be paired with investments in video and portal infrastructure.^38^ From an implementation perspective, learning health systems could leverage EHR-derived engagement phenotypes for continuous monitoring dashboards to identify digitally underserved groups, and proactively offer multilingual digital navigation support, portal activation during in-person visits, video visit onboarding, and community-based digital literacy training programs. Importantly, advancing digital health equity requires addressing barriers across multiple levels, including policies, systems, communities, individuals, and the technology itself.^39^

### Limitations

This study has several limitations. Clustering used total encounters over two years rather than longitudinal trajectories, so temporal changes were not captured. Cluster analysis is exploratory and findings should not be interpreted causally, and cluster labels are descriptive summaries of empirically derived patterns which may influence interpretation. HbA1c outcomes were derived from an 18-month window to reduce missingness, but lower engaged patients had more missing data, potentially biasing results toward the null. Dichotomizing HbA1c improved clinical interpretability but masked the magnitude of change. Finally, analyses were limited to two urban San Francisco systems, and findings may not be generalizable to rural settings, systems with different digital infrastructure, or reimbursement environments. Despite these limitations, this study leverages comprehensive, multimodal encounter data and provides one of the first cross-system, phenotype-based analyses of hybrid care engagement in T2DM.

## Conclusion

In two urban health systems, we identified four hybrid care engagement phenotypes per system that capture how adults with T2DM combine in-person, telephone, video, and portal messaging modalities. Phenotype membership differed markedly by system context and sociodemographic characteristics, reflecting structural and digital access inequities. Digitally forward phenotypes showed modest, system-specific associations with glycemic control among patients with uncontrolled diabetes, though baseline glycemic status remained the primary predictor of outcomes. These findings underscore that equitable hybrid care requires not only access to digital modalities but also culturally and linguistically tailored support to sustain engagement. Operationalizing these phenotypes with routinely collected EHR data may enable learning health systems to monitor hybrid care engagement, target outreach to underserved groups, and advance digital health equity.

## Supplementary Material

ocag063_Supplementary_Data

## Data Availability

The data underlying this article will be shared on reasonable request to the corresponding author.
