# Supplementary material for: Hybrid care engagement phenotypes and glycemic outcomes in diabetes: a cluster analysis across two health systems
Source: J Am Med Inform Assoc. 2026 May 6;33(7):1271–83. doi: 10.1093/jamia/ocag063 (PMC13317988; doi:10.1093/jamia/ocag063)
Supplement: ocag063_Supplementary_Data [file ocag063_supplementary_data.docx]

Supplementary Materials

# Table of Contents

## S1. Supplementary Methods

# S1.1 Data Preparation

# S1.2 Cluster Derivation & Evaluation

# S1.3 Phenotype Labeling

# S1.4 Logistic Regression Model Specifications

# S1.5 Model Diagnostics

# S1.6 Sensitivity Analyses

# S1.7 Software

## S2. Supplementary Tables

S2.1 Table S1. Eligibility criteria

S2.2 Table S2. Encounter types and definitions
S2.3 Table S3. Provider types and definitions
S2.4 Table S4. Sociodemographic and clinical variables and coding
S2.5 Table S5. Descriptives of Baseline and Last HbA1c Values by Phenotype (UCSF)
S2.6 Table S6. Descriptives of Baseline and Last HbA1c Values by Phenotype (SFHN)
S2.7 Table S7. Analytic sample derivation for logistic regression models
S2.8 Table S8. Total encounters over 2 years by modality and health system
S2.9 Table S9. Average silhouette widths for cluster solutions (k = 2–10)
S2.10 Table S10. Differences in Patient Characteristics Across Engagement Phenotypes (UCSF)
S2.11 Table S11. Differences in Patient Characteristics Across Engagement Phenotypes (SFHN)
S2.12 Table S12. Comparative Profiles of Phenotypes Across Health Systems
S2.13 Table S13. Multivariable logistic regression results (main-effects)
S2.14 Table S14. Predicted probabilities & Tukey contrasts (UCSF)
S2.15 Table S15. Predicted probabilities & Tukey contrasts (SFHN)

## S3. Supplementary Figures

S3.1 Figure S1. Quarterly mean encounters per patient by modality and health system over the 2-year study period

S3.2 Figure S2. Standardized encounter rates by modality
S3.3 Figure S3. Silhouette widths for k = 2–10

S3.4 Figure S4. ROC Curve for UCSF Interaction Model
S3.5 Figure S5. Calibration Plot for UCSF Interaction Model

S3.6 Figure S6. ROC Curve for SFHN Interaction Model
S3.7 Figure S7. Calibration Plot for SFHN Interaction Model

## **S1. Supplementary Methods**

### S1.1 Data Preparation

Encounter data were extracted for all adults meeting the eligibility criteria (Table S1). For each patient, we calculated two-year total for each of the five encounter modalities: in-person, video, telephone, portal messages, and unscheduled phone calls (definitions in Table S2). Zero values were retained to indicate modality non-use and to preserve interpretability of sparse engagement patterns. Encounter counts were highly skewed and zero-inflated; therefore, counts were transformed using log(count + 1) to stabilize variance and mitigate the influence of extreme outliers. Transformed features were subsequently z-score normalized to ensure comparability across modalities with different utilization ranges.

### S1.2 Cluster Derivation & Evaluation

We used k-medoids clustering (PAM) with Manhattan distance due to robustness to outliers and sparse, zero-inflated count data [1, 2]. Clustering was performed separately by health system to account for system-level differences. To ensure stability and reproducibility, we ran the algorithm with 25 random starts using a fixed random seed (set.seed(42)). We evaluated k = 2–10 using silhouette widths, compactness, and interpretability. Results appear in Table S9 and Figure S3.

### S1.3 Phenotype Labeling

We labeled phenotypes using a rule-based procedure informed by cluster-level descriptive statistics. For each cluster, we evaluated (1) the relative magnitude of encounter use across the five modalities, (2) the dominant modality or modality group, (3) the breadth of modality engagement, and (4) the relative balance of digital vs. traditional modalities. Labels reflected the dominant engagement pattern, and cluster summaries were used to validate interpretability.

### S1.4 Logistic Regression Model Specifications

We summarized baseline and follow-up HbA1c values, and their change, by engagement phenotype and baseline glycemic control status within each health system (Tables S5–S6). Logistic regression models were estimated for each health system with LastA1c (1 = controlled ≤8%, 0 = uncontrolled >8%) as the binary outcome:

$$\text{logit}(P(\text{LastA1c}_{i}=1))=\beta_{0}+\beta_{1}\text{Phenotype}_{i}+\beta_{2}\text{BaselineHbA1c}_{i}+\beta_{3}X_{i}.$$

where $X_{i}$includes age group, sex, race/ethnicity, insurance, preferred language, and Charlson Comorbidity Index category. To assess effect modification by baseline glycemic status, we fit an interaction model that included phenotype × baseline HbA1c terms:

$$\text{logit}(P(\text{LastA1c}_{i}=1))=\beta_{0}+\beta_{1}\text{Phenotype}_{i}+\beta_{2}\text{BaselineHbA1c}_{i}+\beta_{3}(\text{Phenotype}_{i}\times\text{BaselineHbA1c}_{i})+\beta_{4}X_{i}.$$

A likelihood ratio test compared the interaction model with the main-effects model to find the best fitting model. Population-marginal predicted probabilities and pairwise contrasts were estimated using the *emmeans* package, which averages model-based predictions over the observed covariate distribution [3]. Pairwise comparisons were adjusted for multiple testing using Tukey’s adjustment. This marginal standardization approach yields population-average contrasts that are more interpretable than conditional estimates at fixed covariate values [4].

### S1.5 Model Diagnostics

### Model fit and performance were assessed using AIC, likelihood ratio tests, AUC, and Hosmer–Lemeshow calibration. Categorical predictors had sufficient cell counts (>100 per outcome). Multicollinearity was evaluated using variance inflation factors (all <2), and influence was assessed using Cook’s distance and leverage/residual diagnostics

### S1.6 Sensitivity Analyses

### A complete-case approach was used due to low missingness (<10%; Table S6). Sensitivity analyses restricted the cohort to patients with baseline and follow-up HbA1c measured within 12 months.

### S1.7 Software

Analyses were conducted in R 4.3.2 on macOS 14.4 using cluster, factoextra, car, emmeans, pROC, and tidyverse.

## **S2. Supplementary Tables**

**Table S1:** **Eligibility criteria**

| **Inclusion Criteria** |
| --- |
| - Adults ≥18 years - Type 2 Diabetes Mellitus (T2DM) - Empaneled in primary care as of 4/1/2019 - Active care receipt defined by:   - At least one encounter with their primary care or endocrinology teams between April 1, 2019 – March 31, 2021   - At least one healthcare interaction (including patient portal messages, phone calls) in the last 12 months of the study period (April 1, 2022 – March 31, 2023) |
| **Exclusion Criteria** |
| - Non-T2DM (pregnancy-related, T1DM) - Death during study period - Lost to follow-up (defined as no touchpoint with health system in last 12 months of study period) |

**Table S2:** **Encounter types and definitions**

| **ENCOUNTER TYPES** | **DEFINITIONS** |
| --- | --- |
| **Scheduled** | |
| In-person | Office Visit, Nurse Only, Social Work, Nutrition |
| Video | Video Visit, Nutrition + video visit or telemedicine, Telemedicine, Video Visit Non-Billable |
| Telephone | Scheduled Telephone Encounter, Nutrition + telephone |
| **Unscheduled** | |
| Message | Patient Message, E-Visit |
| Outreach calls | Unscheduled telephone calls with any member of care team |

**Table S3: Provider types and definitions**

| **PROVIDER TYPES** | **DEFINITIONS** |
| --- | --- |
| Billable | Physician, Nurse Practitioner, Resident, Physician Assistant, Fellow |
| Registered Nurse | Registered Nurse |
| Other | Health Worker, Medical Assistant, Pharmacist, Social Worker, Eligibility Worker, Clerk, Resource, Registered Dietician, Case Manager, Health Care Navigator, Therapist, Psychologist, Technician, Licensed Vocational Nurse, Hospital Assistant, Medical Student, Public Health Nurse, Primary Care Location, Coordinator, IP Patient Navigator, Pharmacy Student, Health Educator, Ophthalmologist, Scribe, Person, APN Student, Athletic Trainer, NULL |

**Table S4:** **Sociodemographic and clinical variables and coding**

| **VARIABLE** | **CODING** | **DEFINITIONS** |
| --- | --- | --- |
| Birth Year | Range: 1920–2001 | Year of birth |
| Age | Range: 20–101 | Age in years (continuous) as of 4/1/2021 |
| Age Group | 1 = <50  2 = 50–65  3 = 65–74  4 = 75+ | Age categories as of 4/1/2021 |
| Sex | 1 = Female  2 = Male  5 = Other/Unknown | Sex assigned at birth |
| Race/Ethnicity | 1 = Non-Hispanic Asian American  2 = Non-Hispanic African American  3 = Hispanic or Latine  4 = Non-Hispanic White  5 = Other/Unknown | Patient-reported race/ethnicity, grouped |
| Preferred Language | 1 = English  2 = Spanish  3 = Chinese  4 = Other/Unknown | Patient-reported preferred language |
| Insurance Type | 1 = Commercial (Private, Worker’s Comp, Medicare Advantage, Multiplan, Vision, Veterans)  2 = Medicaid (Medi-Cal, Medicaid, SF Health Plan)  3 = Medicare  4 = Uninsured (Patient Pay, Uninsured, Anchor Study, UHC HRSA)  5 = Healthy Workers | Insurance as of 4/1/2021 |
| Health System | 1 = SFHN  2 = UCSF | Care delivery system |
| Neighborhood Socioeconomic Status (nSES) | 1 = Q1 (lowest)  2 = Q2  3 = Q3  4 = Q4  5 = Q5 (highest) | Quintiles scaled to 9 Bay Area counties, based on most recent address as of 4/1/2021 |
| Patient Portal Activation Status | 0 = Inactive  1 = Active | MyChart portal status as of 4/1/2021 |
| Charlson Comorbidity Index | Range: 0–16 | Based on active diagnoses between 4/1/2020–3/31/2021 |
| Baseline A1c Control | 0 = Uncontrolled (>8%)  1 = Controlled (≤8%) | Most recent A1c as of 4/1/2021 (Oct 2019–Mar 2021) |
| Follow-up A1c Control | 0 = Uncontrolled (>8%)  1 = Controlled (≤8%) | Most recent A1c as of 3/31/2023 (Oct 2021–March 2023) |

## **Table S5. Mean (SD) Baseline and Last HbA1c Values by Engagement Phenotype and Baseline HbA1c Control Status (UCSF analytic sample, N=3,241)**

| **Baseline A1c Status** | **n (%)** | **Baseline A1c Mean (SD)** | **Last A1c Mean (SD)** | **Δ (Last – Baseline)** |
| --- | --- | --- | --- | --- |
| **Controlled (≤8%)** | | | | |
| Low Digital | 786 (31%) | 6.7 (0.6) | 6.8 (1.2) | +0.2 |
| Digital Leaning | 724 (28%) | 6.7 (0.7) | 6.9 (1.2) | +0.2 |
| Traditional High Utilizers | 283 (11%) | 6.7 (0.8) | 6.8 (1.3) | +0.1 |
| Digitally Engaged Multimodal | 758 (30%) | 6.7 (0.7) | 6.8 (1.2) | +0.2 |
| **Uncontrolled (>8%)** | | | | |
| Low Digital | 171 (25%) | 9.5 (1.5) | 8.6 (1.7) | −0.9 |
| Digital Leaning | 132 (19%) | 9.3 (1.3) | 8.3 (1.6) | −1.0 |
| Traditional High Utilizers | 114 (17%) | 9.9 (1.8) | 8.9 (1.9) | −1.0 |
| Digitally Engaged  Multimodal | 273 (40%) | 9.7 (1.5) | 8.5 (1.8) | −1.2 |

*Note.* n (%) indicates the number and proportion within each baseline control category.

## **Table S6. Mean (SD) Baseline and Last HbA1c Values by Engagement Phenotype and Baseline HbA1c Control Status (SFHN analytic sample, N=5,751)**

| **Baseline A1c Status** | **n (%)** | **Baseline A1c Mean (SD)** | **Last A1c Mean (SD)** | **Δ (Last – Baseline)** |
| --- | --- | --- | --- | --- |
| **Controlled (≤8%)** | | | | |
| Low Digital | 681 (17%) | 6.8 (0.6) | 7.2 (1.6) | +0.4 |
| Telephone Reliant | 1470 (37%) | 6.8 (0.6) | 7.1 (1.2) | +0.3 |
| Traditional High Utilizers | 1204 (31%) | 6.8 (0.7) | 7.1 (1.3) | +0.3 |
| Digitally Engaged Multimodal | 579 (15%) | 6.8 (0.7) | 7.0 (1.3) | +0.2 |
| **Uncontrolled (>8%)** | | | | |
| Low Digital | 278 (15%) | 9.9 (1.6) | 9.0 (2.1) | -0.8 |
| Telephone Reliant | 552 (30%) | 9.9 (1.7) | 9.1 (2.2) | -0.7 |
| Traditional High Utilizers | 717 (40%) | 10.0 (1.6) | 8.8 (2.0) | -1.1 |
| Digitally Engaged Multimodal | 270 (15%) | 9.8 (1.7) | 8.6 (2.0) | -1.2 |

**Table S7.** **Analytic sample derivation for logistic regression models**

| **STEP** | **UCSF (N=3,860)** | **SFHN (N=6,811)** |
| --- | --- | --- |
| Initial eligible cohort | 3,860 | 6,811 |
| **Missing outcome (HbA1c)** | **325 (8.4%)** | **571 (8.4%)** |
| Follow-up only missing | 204 (5.3%) | 426 (6.3%) |
| Both baseline & follow-up missing | 121 (3.1%) | 145 (2.1%) |
|  |  |  |
| **Missing predictors** | **294 (7.6%)** | **489 (7.2%)** |
| Baseline HbA1c missing* | 250 (6.5%) | 441 (6.5%) |
| Insurance missing | 31 (0.8%) | — |
| Charlson score missing | 13 (0.3%) | 21 (0.3%) |
| Sex missing | — | 1 (0.0%) |
| Implausible values (commercial insurance at SFHN) | — | 26 (0.4%) |
| **Final analytic sample for regression models** | **3,241 (83.9%)** | **5,751 (84.4%)** |

**Baseline HbA1c missing includes patients with both baseline and follow-up HbA1c missing, which also appear in the outcome-missing category.*

**Table S8. Total encounters over 2 years by modality and health system**

|  | **UCSF (N = 3,860)** | | **SFHN (N = 6,811)** | |
| --- | --- | --- | --- | --- |
| Care modality | Total encounters | Encounters per 100 patients | Total encounters | Encounters per 100 patients |
| In-person visits | 20118 | 521.2 | 48146 | 706.9 |
| Video visits | 9518 | 246.6 | 352 | 5.2 |
| Telephone visits | 1720 | 44.6 | 22652 | 332.6 |
| Portal messages | 43578 | 1129.0 | 12554 | 184.3 |
| Unscheduled telephone calls | 40627 | 1052.5 | 90799 | 1333.1 |

**Table S9. Average silhouette widths for cluster solutions (k = 2-10)**

| **k** | **UCSF (N = 3,860)** | **SFHN (N = 6,811)** |
| --- | --- | --- |
| 2 | 0.25 | 0.25 |
| 3 | 0.20 | 0.18 |
| 4 | 0.22 | 0.22 |
| 5 | 0.20 | 0.22 |
| 6 | 0.19 | 0.19 |
| 7 | 0.18 | 0.21 |
| 8 | 0.18 | 0.21 |
| 9 | 0.17 | 0.20 |
| 10 | 0.18 | 0.21 |

*.*

**Table S10. Differences in Patient Characteristics Across Engagement Phenotypes (UCSF; N = 3,860)**

|  | **Digitally Engaged Multimodal** | **Traditional High Utilizers** | **Digital Leaning** | **Low Digital** | **P-value** |
| --- | --- | --- | --- | --- | --- |
| **n (%)** | 1124 (29%) | 457 (12%) | 1054 (27%) | 1225 (32%) |  |
|  |  |  |  |  |  |
| **Age (years, mean ± SD)** | 66.1 ± 13.8 | 71.2 ± 12.1 | 63.2 ± 14.0 | 68.0 ± 13.1 | < 0.001 |
| **Sex (%)** | | | | | < 0.001 |
| Female | 670 (59.6) | 290 (63.5) | 524 (49.7) | 607 (49.6) |  |
| Male | 454 (40.4) | 167 (36.5) | 530 (50.3) | 618 (50.4) |  |
| **Race/Ethnicity (%)** |  |  |  |  | < 0.001 |
| Hispanic/Latine | 156 (13.9) | 56 (12.3) | 111 (10.5) | 157 (12.8) |  |
| NH Asian American | 393 (35.0) | 130 (28.4) | 406 (38.5) | 599 (48.9) |  |
| NH African American | 179 (15.9) | 127 (27.8) | 81 (7.7) | 140 (11.4) |  |
| NH White | 297 (26.4) | 96 (21.0) | 339 (32.2) | 209 (17.1) |  |
| Other/Unknown | 99 (8.8) | 48 (10.5) | 117 (11.1) | 120 (9.8) |  |
| **Preferred Language (%)** | | | | | < 0.001 |
| English | 945 (84.1) | 345 (75.5) | 944 (89.6) | 920 (75.1) |  |
| Spanish | 29 (2.6) | 22 (4.8) | 14 (1.3) | 47 (3.8) |  |
| Chinese | 70 (6.2) | 49 (10.7) | 43 (4.1) | 134 (10.9) |  |
| Other/Unknown | 80 (7.1) | 41 (9.0) | 53 (5.0) | 124 (10.1) |  |
| **Insurance (%)** | | | | | < 0.001 |
| Commercial | 484 (43.1) | 86 (18.8) | 596 (56.5) | 521 (42.6) |  |
| Medicaid | 150 (13.3) | 89 (19.5) | 77 (7.3) | 178 (14.5) |  |
| Medicare | 479 (42.6) | 279 (61.1) | 369 (35.0) | 512 (41.8) |  |
| Unknown/Missing | 11(1.0) | 3 (0.7) | 12 (1.1) | 13 (1.1) |  |
| **Neighborhood SES (%)** | | | | | < 0.001 |
| 1 (lowest) | 51 (4.5) | 47 (10.3) | 23 (2.2) | 58 (4.7) |  |
| 2 | 84 (7.5) | 35 (7.7) | 43 (4.1) | 88 (7.2) |  |
| 3 | 145 (12.9) | 57 (12.5) | 119 (11.3) | 146 (11.9) |  |
| 4 | 274 (24.4) | 118 (25.8) | 239 (22.7) | 287 (23.4) |  |
| 5 (highest) | 532 (47.3) | 167 (36.5) | 564 (53.5) | 590 (48.2) |  |
| Missing | 38 (3.4) | 33 (7.2) | 66 (6.3) | 55 (4.5) |  |
| **Patient Portal Status (%)** | | | | | < 0.001 |
| Active | 1084 (96.4) | 292 (63.9) | 1033 (98.0) | 974 (79.6) |  |
| Inactive | 40 (3.6) | 165 (36.1) | 21 (2.0) | 250 (20.4) |  |
| **Charlson score**  (mean ± SD) | 2.2 ± 2.6 | 2.3 ± 2.5 | 1.4 ± 2.1 | 1.2 ± 1.9 | < 0.001 |
| **Baseline A1c Control (%)** | | | | | < 0.001 |
| Controlled (≤ 8%) | 794 (70.6) | 304 (66.5) | 800 (75.9) | 892 (72.9) |  |
| Uncontrolled (> 8%) | 278 (24.7) | 115 (25.2) | 151 (14.3) | 200 (16.3) |  |
| Missing | 52 (4.6) | 38 (8.3) | 103 (9.8) | 132 (10.8) |  |

*P-values represent global tests of differences across phenotypes (chi-square for categorical variables, Kruskal-Wallis tests for continuous variables.*

**Table S11. Differences in Patient Characteristics Across Engagement Phenotypes (SFHN; N = 6,811)**

|  | **Digitally Engaged Multimodal** | **Traditional High Utilizers** | **Telephone Reliant** | **Low Digital** | **P-value** |
| --- | --- | --- | --- | --- | --- |
| **n (%)** | 939 (14%) | 2094 (31%) | 2396 (35%) | 1382 (20%) |  |
|  |  |  |  |  |  |
| **Age (years, mean ± SD)** | 59.4 ± 13.1 | 63.6 ± 11.0 | 61.2 ± 11.3 | 61.5 ± 11.7 | < 0.001 |
| **Sex (%)** | | | | | < 0.001 |
| Female | 464 (49.4) | 1164 (55.6) | 1303 (54.4) | 650 (47.0) |  |
| Male | 475 (50.6) | 930 (44.4) | 1092 (45.6) | 732 (53.0) |  |
| **Race/Ethnicity (%)** |  |  |  |  | < 0.001 |
| Hispanic/Latine | 275 (29.3) | 817 (39.0) | 740 (30.9) | 498 (36.0) |  |
| NH Asian American | 263 (28.0) | 545 (26.0) | 1137 (47.5) | 449 (32.5) |  |
| NH African American | 136 (14.5) | 438 (20.9) | 238 (9.9) | 215 (15.6) |  |
| NH White | 211 (22.5) | 185 (8.8) | 191 (8.0) | 135 (9.8) |  |
| Other/Unknown | 54 (5.8) | 109 (5.2) | 90 (3.8) | 85 (6.2) |  |
| **Preferred Language (%)** | | | | | < 0.001 |
| English | 607 (64.6) | 927 (44.3) | 778 (32.5) | 619 (44.8) |  |
| Spanish | 160 (17.0) | 703 (33.6) | 627 (26.2) | 411 (29.7) |  |
| Chinese | 69 (7.3) | 307 (14.7) | 762 (31.8) | 186 (13.5) |  |
| Other/Unknown | 103 (11.0) | 157 (7.5) | 229 (9.6) | 166 (12.0) |  |
| **Insurance (%)** | | | | | < 0.001 |
| Commercial | 3 (0.3) | 17 (0.8) | 12 (0.5) | 11 (0.8) |  |
| Medicaid | 354 (37.7) | 757 (36.2) | 790 (33.0) | 531 (38.4) |  |
| Medicare | 349 (37.2) | 911 (43.5) | 669 (27.9) | 434 (31.4) |  |
| Healthy Workers | 139 (14.8) | 140 (6.7) | 531 (22.2) | 162 (11.7) |  |
| Uninsured | 78 (8.3) | 236 (11.3) | 347 (14.5) | 198 (14.3) |  |
| Unknown/Missing | 16 (1.7) | 33 (1.6) | 47 (2.0) | 46 (3.3) |  |
| **Neighborhood SES (%)** | | | | | < .001 |
| 1 (lowest) | 87 (9.3) | 319 (15.2) | 262 (10.9) | 256 (18.5) |  |
| 2 | 100 (10.6) | 277 (13.2) | 301 (12.6) | 142 (10.3) |  |
| 3 | 185 (19.7) | 487 (23.3) | 603 (25.2) | 283 (20.5) |  |
| 4 | 213 (22.7) | 440 (21.0) | 507 (21.2) | 315 (22.8) |  |
| 5 (highest) | 328 (34.9) | 495 (23.6) | 658 (27.5) | 344 (24.9) |  |
| Missing | 26 (2.8) | 76 (3.6) | 65 (2.7) | 42 (3.0) |  |
| **Patient Portal Status (%)** | | | | | < 0.001 |
| Active | 675 (71.9) | 220 (10.5) | 386 (16.1) | 182 (13.2) |  |
| Inactive | 264 (28.1) | 1874 (89.5) | 2010 (83.9) | 1200 (86.8) |  |
| **Charlson score**  (mean ± SD) | 1.3 ± 1.9 | 1.4 ± 1.9 | 0.7 ± 1.4 | 0.8 ± 1.6 | < 0.001 |
| **Baseline A1c Control (%)** | | | | | < 0.001 |
| Controlled (≤ 8%) | 599 (63.8) | 1242 (59.3) | 1587 (66.2) | 839 (60.7) |  |
| Uncontrolled (> 8%) | 278 (29.6) | 746 (35.6) | 604 (25.2) | 345 (25.0) |  |
| Missing | 62 (6.6) | 106 (5.1) | 205 (8.6) | 198 (14.3) |  |

**Table S12. Comparative Profiles of Hybrid Care Engagement Phenotypes Across Health Systems**

| **Phenotype** | **Academic System**  **Key Characteristics** | **Safety-Net System**  **Key Characteristics** | **Similarities & Differences** |
| --- | --- | --- | --- |
| Digitally Engaged Multimodal | Younger (mean 66); highest Hispanic patients (14%), more English speakers, high commercial/Medicare coverage; 96% portal activation; 72% in top two nSES quintiles, mean CCI 2.2 | Youngest (mean 59); highest non-Hispanic White (23%) and English speaking; high Medicaid/Medicare insured; highest nSES (58% in top two quintiles); highest portal activation (72%); CCI mean 1.3 | Sim: Both are younger, English speaking, high commercial/Medicare insured, higher nSES, high portal activation, CCI second highest among system.  Diff: UCSF has more Hispanic patients, SFHN has more White patients, youngest age group. |
| Traditional High Utilizers | Oldest (mean 71); highest comorbidity (2.3); more female, highest African American; higher Spanish and Chinese language speakers,  lowest nSES; lowest portal activation (64%); highest Medicaid/Medicare; highest uncontrolled A1c (25.2) | Oldest (mean 64); highest comorbidity (mean 1.4); highest African American (21%) and Hispanic (39%); highest Spanish speaking; lowest portal activation (11%); highest Medicare insured; low nSES 28.4% in bottom 2 quintiles; highest (36%) uncontrolled HbA1c | Sim: Both oldest and sickest with highest comorbidity; highest African American patients, higher Spanish speakers, lowest portal activation, highest Medicare insured, lower nSES, highest uncontrolled A1c  Diff: UCSF has more Chinese speakers and Medicaid insure, SFHN has more Hispanic patients. |
| Digital Leaning (UCSF-only) | Youngest (mean 63); highest non-Hispanic White and English speakers (90%); highest commercial insurance (57%); nearly all portal activated (98%); 76% in top 2 nSES quintiles; highest (76%) controlled HbA1c, lower CCI (mean 1.4) | N/A | N/A |
| Telephone Reliant (SFHN-only) | N/A | Moderate age (mean 61); highest Asian American (48%) and Chinese-speaking (32%); highest uninsured (15%) and Healthy Workers (22%); 49% in top 2 nSES, low portal activation (16%); highest (66%) controlled HbA1c, lowest CCI (mean 0.7) | N/A |
| Low Digital | Older (mean 68); highest Asian American (49%); 11% Chinese language preference; lower portal activation (80%); most Commercial/Medicare insured; 72% in top two nSES quintiles; lowest comorbidity burden (mean 1.2) | Moderate age (mean 62); higher Hispanic (36%) and Spanish speaking (30%); lowest SES (19% bottom quintile); highest Medicaid (38%); lower portal activation (13%); highest missing HbA1c (14%), lower CCI (0.8) | Sim: Skewed older, non-English language speakers, lower portal activation, lower CCI.  Diff: UCSF has more Asian American and Chinese speakers, Commercial/Medicare insured, high nSES, lowest CCI.  SFHN – Hispanic, Spanish speakers, lowest nSES, highest Medicaid, highest missing A1c. |

**Table S13.** **Multivariable logistic regression of glycemic control at follow-up (main-effects model) across both health systems**

|  | **Academic (UCSF)** | | **Safety-Net (SFHN)** | |
| --- | --- | --- | --- | --- |
| **Predictor** | **aOR (95% CI)** | **p-value** | **aOR (95% CI)** | **p-value** |
| **Engagement phenotype** (ref = Low Digital) | | | | |
| Digitally Engaged Multimodal | 1.02 (0.78–1.32) | 0.89 | 1.38 (1.09–1.76) | **0.008** |
| Traditional High Utilizers | 0.77 (0.55–1.08) | 0.13 | 0.92 (0.75–1.12) | 0.40 |
| Digital Leaning | 1.00 (0.75–1.32) | 0.98 | — | — |
| Telephone Reliant | — | — | 1.06 (0.87–1.29) | 0.59 |
| **Baseline HbA1c** (ref = Controlled) | | | | |
| Uncontrolled | 0.11 (0.09–0.13) | **<0.001** | 0.14 (0.12–0.16) | **<0.001** |
| **Age category** (ref = <50 years) | | | | |
| 50–64 | 1.51 (1.11–2.06) | **0.009** | 1.60 (1.32–1.94) | **<0.001** |
| 65–74 | 2.07 (1.47–2.92) | **<0.001** | 2.10 (1.66–2.65) | **<0.001** |
| 75+ | 1.70 (1.18–2.44) | **0.004** | 2.13 (1.58–2.88) | **<0.001** |
| **Sex** (ref = Male) | | | | |
| Female | 1.07 (0.87–1.31) | 0.52 | 1.14 (1.00–1.30) | 0.058 |
| **Race/Ethnicity** (ref = Non-Hispanic White) | | | | |
| Hispanic/Latine | 0.96 (0.67–1.38) | 0.81 | 1.13 (0.83–1.54) | 0.44 |
| NH Asian American | 1.17 (0.89–1.54) | 0.26 | 1.33 (1.02–1.75) | **0.037** |
| NH African American | 0.92 (0.66–1.29) | 0.62 | 1.47 (1.11–1.94) | **0.006** |
| Other | 0.95 (0.67–1.37) | 0.79 | 1.26 (0.89–1.81) | 0.20 |
| **Preferred language** (ref = English) | | | | |
| Chinese | 1.16 (0.75–1.82) | 0.51 | 1.20 (0.92–1.57) | 0.18 |
| Spanish | 1.14 (0.61–2.24) | 0.69 | 0.67 (0.52–0.87) | **0.003** |
| Other/Unknown | 0.82 (0.57–1.21) | 0.32 | 0.95 (0.73–1.25) | 0.73 |
| **Insurance** (ref = Commercial UCSF/ Medicare SFHN) | | | | |
| Medicaid | 0.86 (0.63–1.17) | 0.34 | 0.89 (0.74–1.07) | 0.20 |
| Medicare | 1.13 (0.88–1.45) | 0.34 | — | — |
| Healthy Workers (SFHN only) | — | — | 1.24 (0.97–1.59) | 0.09 |
| Uninsured/Unknown | — | — | 1.10 (0.86–1.41) | 0.45 |
| **Charlson Comorbidity** **Index** (ref = 0) | | | | |
| 1–2 | 1.21 (0.96–1.52) | 0.11 | 1.06 (0.91–1.23) | 0.47 |
| 3–4 | 1.18 (0.87–1.61) | 0.30 | 1.24 (0.96–1.61) | 0.10 |
| 5+ | 1.36 (0.95–1.97) | 0.095 | 1.11 (0.82–1.51) | 0.52 |

Notes: Results shown are from the main-effects logistic regression model (reference = Low Digital). Bold indicates p < .05.

**Table S14.** **Predicted probability of follow-up HbA1c control (≤8%) by engagement phenotype and baseline HbA1c control status for Academic health system (UCSF), with pairwise Tukey-adjusted contrasts (population-marginal estimates)**

| **Baseline HbA1c** | **Phenotype** | **Predicted Probability (%)** | **95% CI** | **Pairwise Contrast** | **aOR** | **95% CI** | **p-value (Tukey)** |
| --- | --- | --- | --- | --- | --- | --- | --- |
| **Controlled**  **(≤8%)** | Low Digital | 90 | 88–92 | Low vs High Traditional | 1.06 | 0.57–1.96 | 0.996 |
|  | Traditional High Utilizers | 90 | 85–93 | Low vs Digital Leaning | 1.21 | 0.78–1.87 | 0.683 |
|  | Digital Leaning | 88 | 86–91 | Low vs Multimodal | 1.06 | 0.68–1.65 | 0.988 |
|  | Digitally Engaged Multimodal | 90 | 87–92 | High Traditional vs Digital Leaning | 1.14 | 0.62–2.11 | 0.946 |
|  |  |  |  | High Traditional vs Multimodal | 1.00 | 0.55–1.83 | >0.999 |
|  |  |  |  | Digital Leaning vs Multimodal | 0.88 | 0.57–1.35 | 0.860 |
| **Uncontrolled**  **(>8%)** | Low Digital | 46 | 38–54 | Low vs High Traditional | 1.54 | 0.79–2.98 | 0.343 |
|  | Traditional High Utilizers | 36 | 27–45 | Low vs Digital Leaning | 0.67 | 0.36–1.25 | 0.360 |
|  | Digital Leaning | 56 | 47–64 | Low vs Multimodal | 0.87 | 0.52–1.46 | 0.901 |
|  | Digitally Engaged Multimodal | 49 | 43–55 | High Traditional vs Digital Leaning | 0.44 | 0.22–0.89 | **0.014** |
|  |  |  |  | High Traditional vs Multimodal | 0.57 | 0.31–1.04 | 0.076 |
|  |  |  |  | Digital Leaning vs Multimodal | 1.29 | 0.74–2.25 | 0.647 |

*Predicted probabilities are population-marginal (sample-weighted) estimated marginal means.

Note: Odds ratios (ORs) represent the odds of HbA1c control for the first phenotype listed relative to the second phenotype (e.g., High Traditional / Digital Leaning). Bold indicates p < .05.

**Table S15.** **Predicted probability of follow-up HbA1c control (≤8%) by engagement phenotype and baseline HbA1c control status for Safety-Net Health System (SFHN), with pairwise Tukey-adjusted contrasts (population-marginal estimates)**

| **Baseline HbA1c** | **Phenotype** | **Predicted Probability (%)** | **95% CI** | **Pairwise Contrast** | **aOR** | **95% CI** | **p (Tukey)** |
| --- | --- | --- | --- | --- | --- | --- | --- |
| **Controlled**  **(≤8%)** | Low Digital | 84 | 81–86 | Low vs Telephone Reliant | 0.84 | 0.60–1.18 | 0.560 |
|  | Telephone Reliant | 86 | 84–88 | Low vs High Traditional | 1.15 | 0.82–1.61 | 0.703 |
|  | Traditional High Utilizers | 82 | 79–84 | Low vs Multimodal | 0.80 | 0.53–1.22 | 0.526 |
|  | Digitally Engaged Multimodal | 87 | 84–89 | Telephone Reliant vs High Traditional | 1.37 | 1.02–1.83 | **0.029** |
|  |  |  |  | Telephone Reliant vs Multimodal | 0.95 | 0.65–1.39 | 0.989 |
|  |  |  |  | High Traditional vs Multimodal | 0.70 | 0.48–1.01 | 0.065 |
| **Uncontrolled**  **(>8%)** | Low Digital | 42 | 36–48 | Low vs Telephone Reliant | 1.11 | 0.74–1.66 | 0.906 |
|  | Telephone Reliant | 40 | 35–44 | Low vs High Traditional | 1.03 | 0.70–1.52 | 0.997 |
|  | Traditional High Utilizers | 41 | 38–45 | Low vs Multimodal | 0.65 | 0.41–1.04 | 0.082 |
|  | Digitally Engaged Multimodal | 53 | 47–59 | Telephone Reliant vs High Traditional | 0.93 | 0.68–1.27 | 0.929 |
|  |  |  |  | Telephone Reliant vs Multimodal | 0.59 | 0.39–0.88 | **0.004** |
|  |  |  |  | High Traditional vs Multimodal | 0.63 | 0.43–0.93 | **0.012** |

*Predicted probabilities are population-marginal (sample-weighted) estimated marginal means.

Note: Odds ratios (ORs) represent the odds of HbA1c control for the first phenotype listed relative to the second phenotype (e.g., Telephone Reliant / High Traditional). Bold indicates p < .05.

## **S3. Supplementary Figures**

**Figure S1. Quarterly mean encounters per patient by modality and health system over the 2-year study period**

*
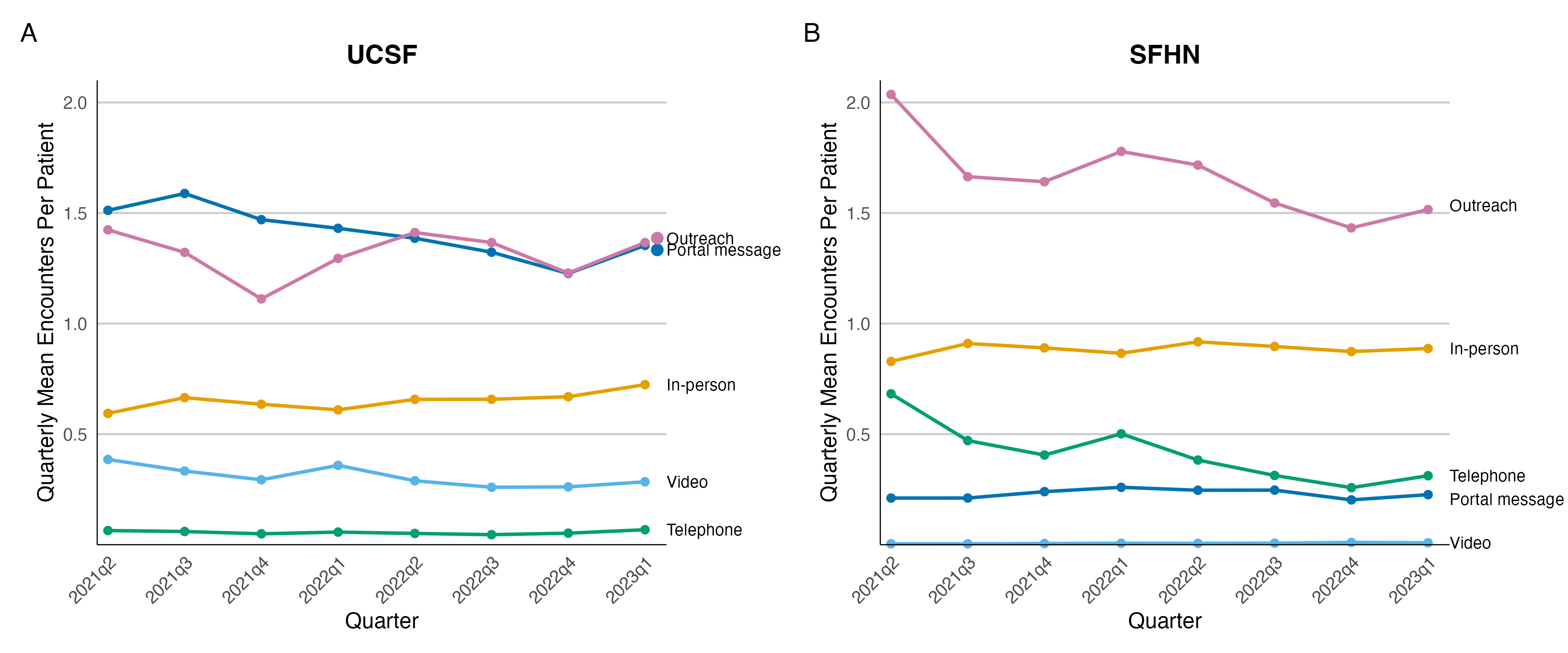
*

**Figure S2. Standardized encounters per 100 patients by modality over study period (UCSF vs. SFHN)**


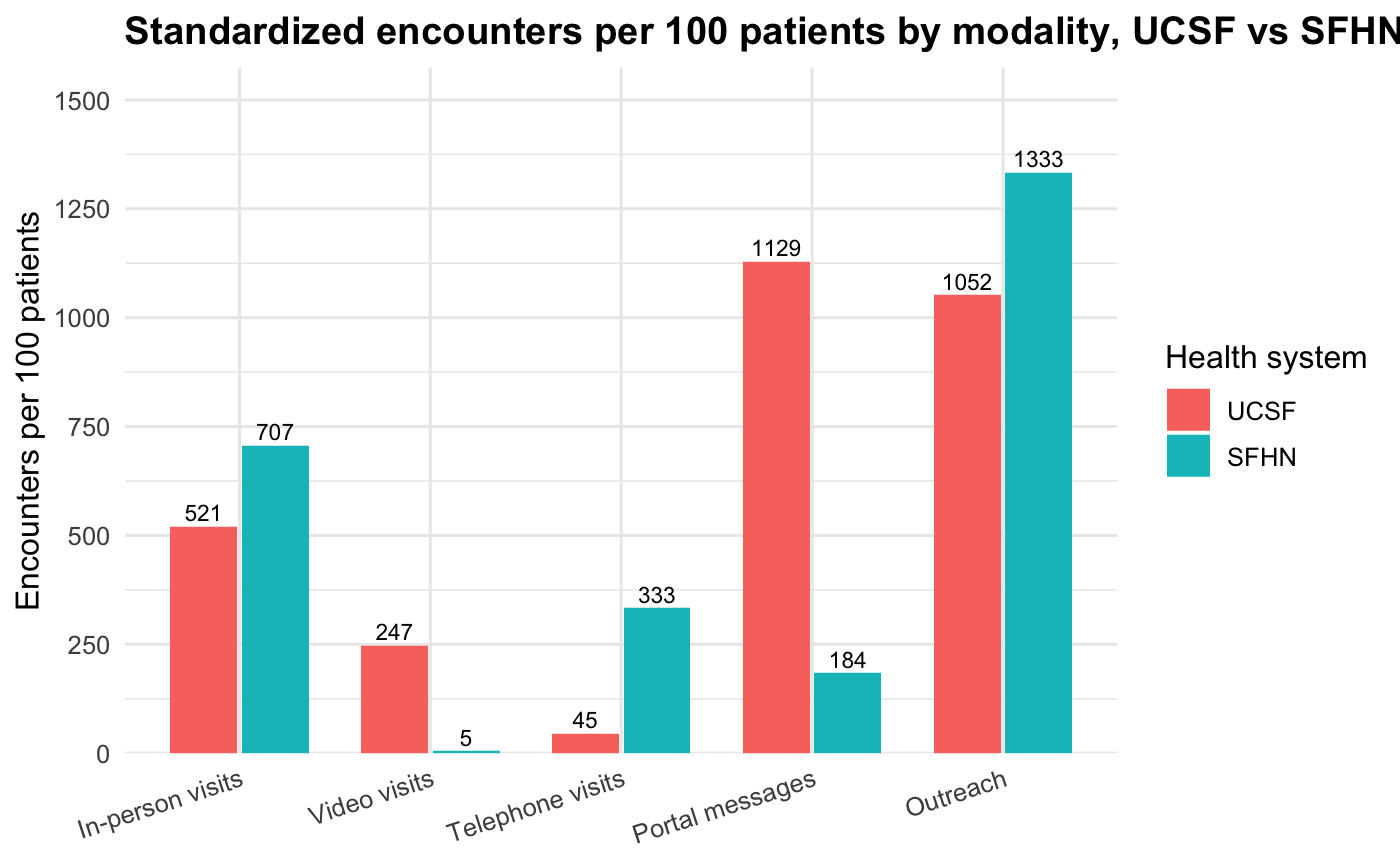


*Standardized encounter rates per 100 patients by modality at UCSF and SFHN, April 2021–March 2023. Rates are normalized to cohort size to enable comparison across systems.*

**Figure S3. Average silhouette widths for k = 2-10 using partitioning around medoids (PAM) with Manhattan distance for UCSF and SFHN.**


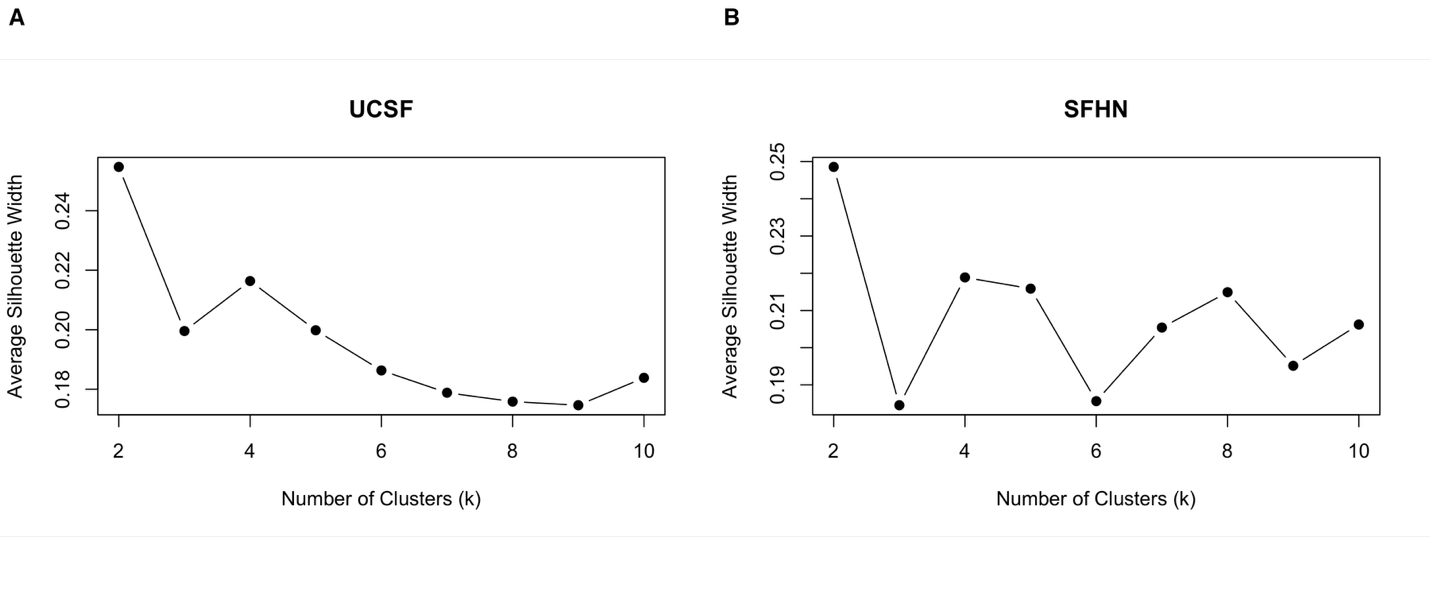


**Figure S4.** **ROC Curve for UCSF Interaction Model (AUC = 0.78)**


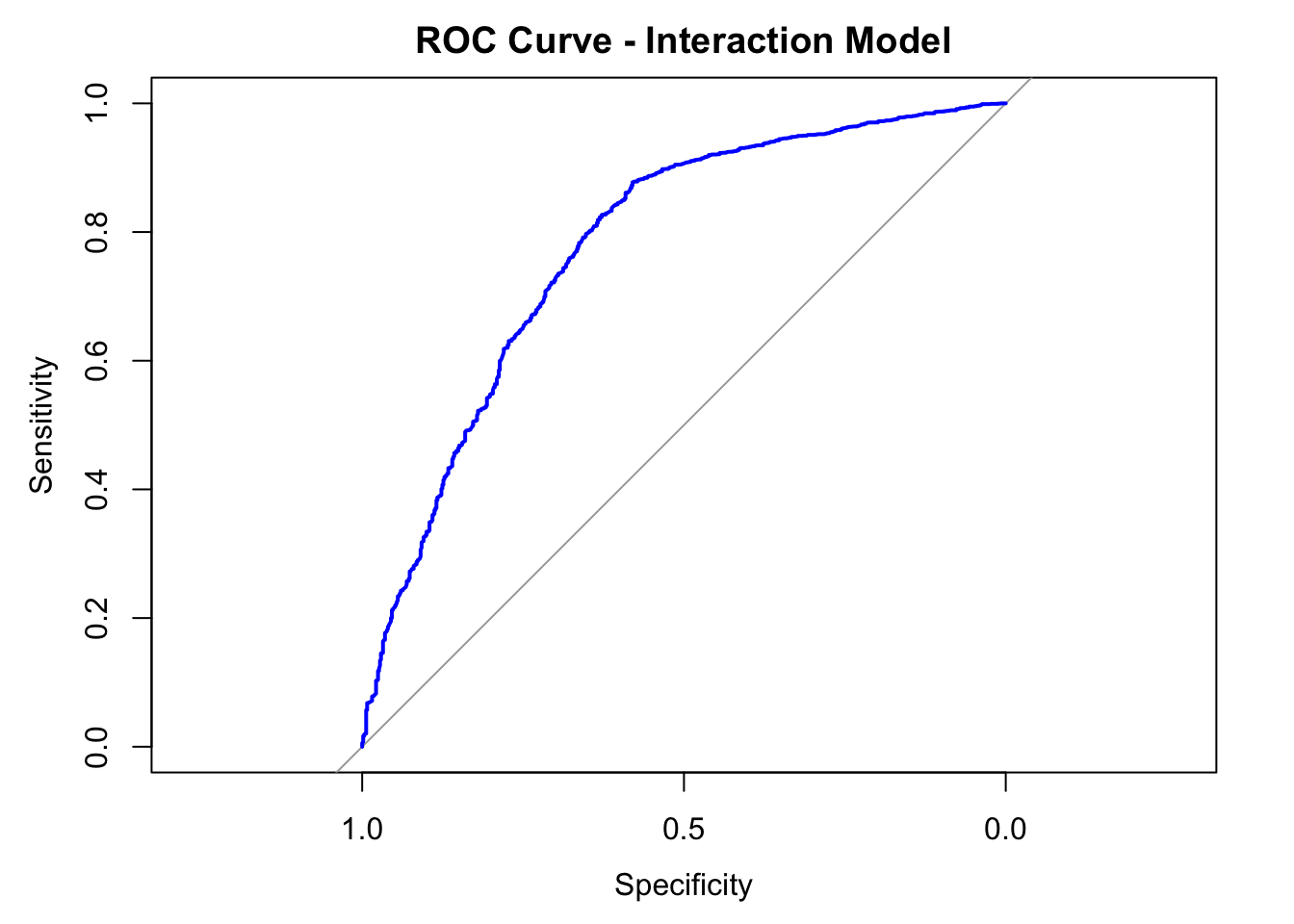


**Figure S5.** **Calibration Plot for UCSF Interaction Model**


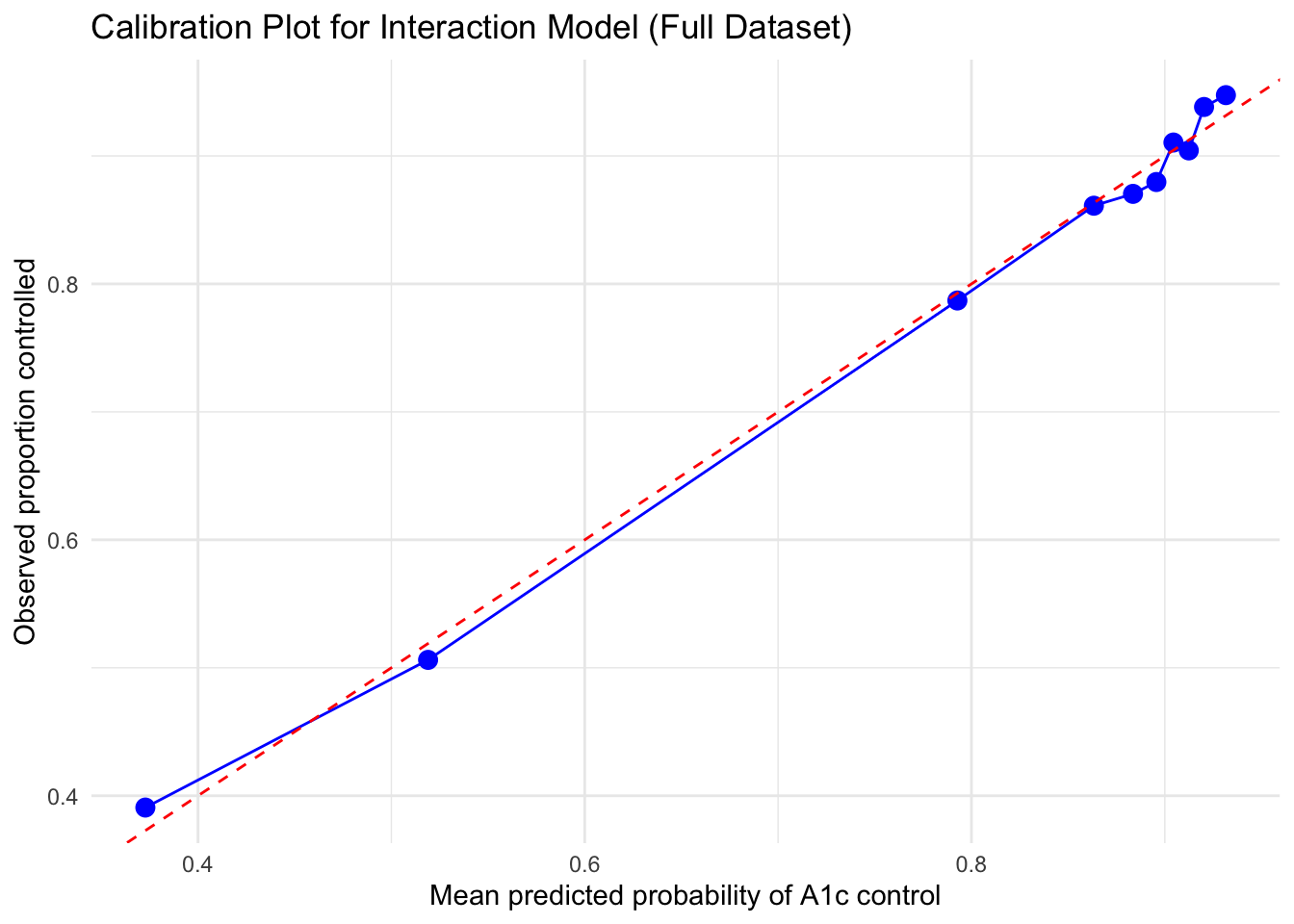


**Figure S6.** **ROC Curve for SFHN Interaction Model (AUC = 0.79)**


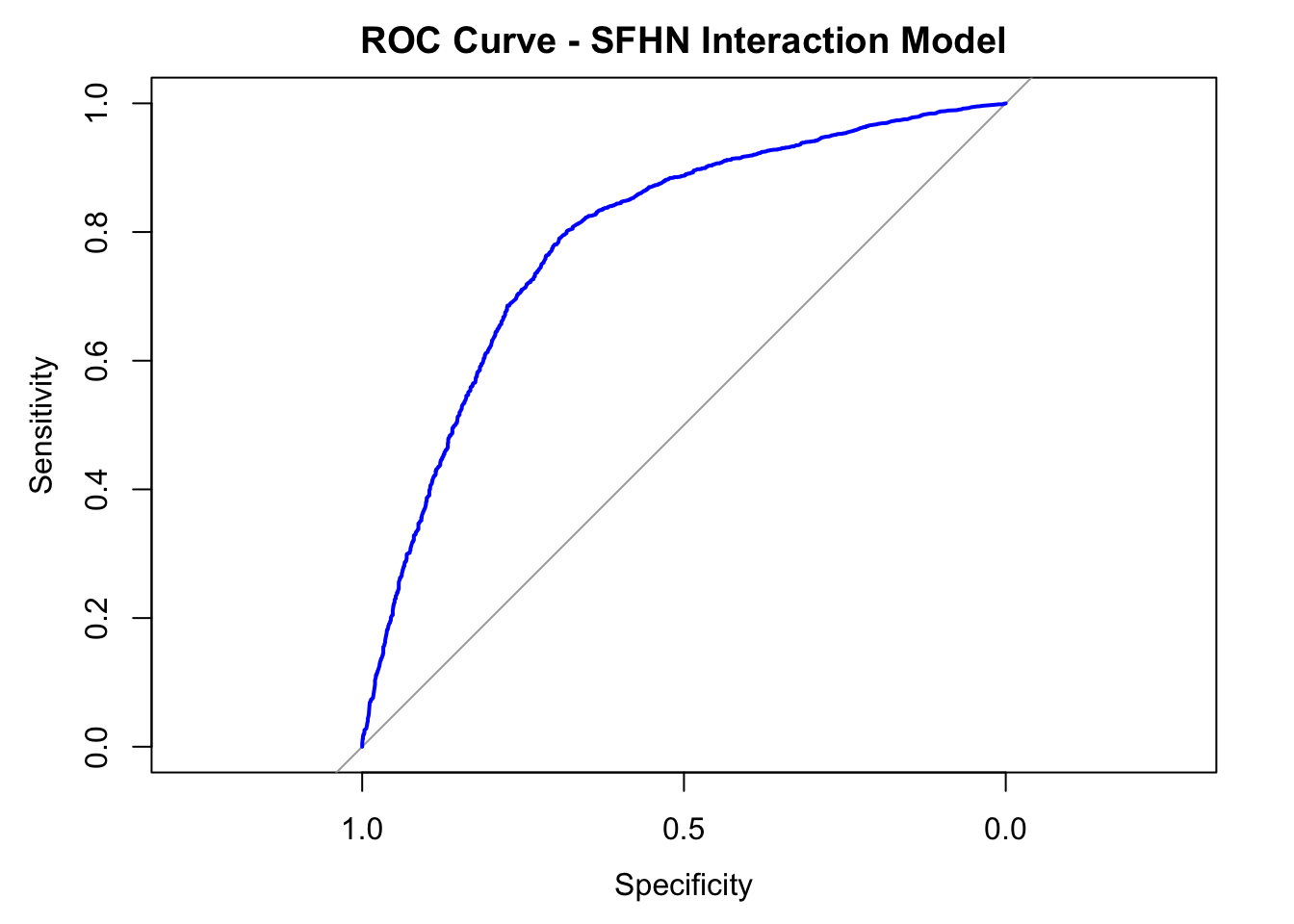


**Figure S7**. **Calibration Plot for SFHN Interaction Model**


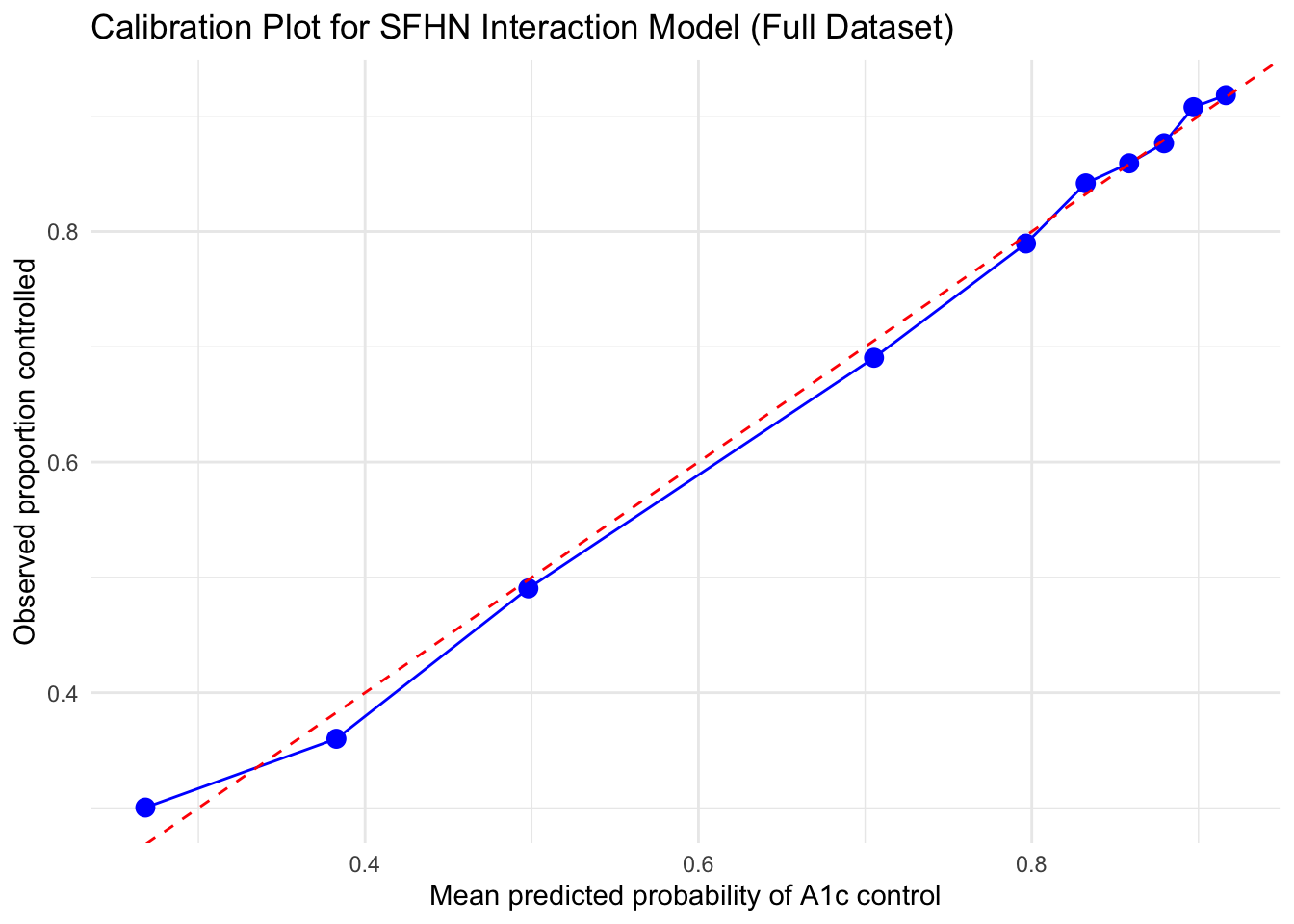


References

1. Park H-S, Jun C-H. A simple and fast algorithm for K-medoids clustering. Expert Systems with Applications. 2009;36(2, Part 2):3336-41.

2. Kaufman L, Rousseeuw P. Finding groups in data: An introduction to cluster analysis: John Wiley & Sons, Inc.; 1990.

3. Lenth R, Banfai, B., Buerkner, P. et al. Estimated Marginal Means, aka Least-Squares Means. 2025.

4. Muller CJ, MacLehose RF. Estimating predicted probabilities from logistic regression: different methods correspond to different target populations. International Journal of Epidemiology. 2014;43(3):962-70.
